# From presentation to paper: Gender disparities in oncological research

**DOI:** 10.1002/ijc.32660

**Published:** 2019-10-11

**Authors:** Willemieke P.M. Dijksterhuis, Charlotte I. Stroes, Wan‐Ling Tan, Suthinee Ithimakin, Antonio Calles, Martijn G.H. van Oijen, Rob H.A. Verhoeven, Jorge Barriuso, Sjoukje F. Oosting, Daniela Kolarevic Ivankovic, Andrew J.S. Furness, Ivana Bozovic‐Spasojevic, Carlos Gomez‐Roca, Hanneke W.M. van Laarhoven

**Affiliations:** ^1^ Department of Medical Oncology Cancer Center Amsterdam, Amsterdam UMC, University of Amsterdam Amsterdam The Netherlands; ^2^ Department of Research and Development Netherlands Comprehensive Cancer Organisation (IKNL) Utrecht The Netherlands; ^3^ Department of Medical Oncology National Cancer Centre Singapore Singapore Singapore; ^4^ Division of Medical Oncology, Department of Medicine Faculty of Medicine Siriraj Hospital, Mahidol University Bangkok Thailand; ^5^ Department of Medical Oncology Hospital General Universitario Gregorio Marañón Madrid Spain; ^6^ Division of Cancer Sciences Manchester Cancer Research Centre, University of Manchester Manchester United Kingdom; ^7^ Department of Medical Oncology The Christie NHS Foundation Trust Manchester United Kingdom; ^8^ Department of Medical Oncology University of Groningen, University Medical Center Groningen Groningen The Netherlands; ^9^ The Royal Marsden NHS Foundation Trust London United Kingdom; ^10^ Institute for Oncology and Radiology of Serbia Belgrade Serbia; ^11^ Institut Universitaire du Cancer de Toulouse (IUCT) Toulouse France

**Keywords:** research, medical oncology, sex, Congresses as topic

## Abstract

Gender disparities in scientific publications have been identified in oncological research. Oral research presentations at major conferences enhance visibility of presenters. The share of women presenting at such podia is unknown. We aim to identify gender‐based differences in contributions to presentations at two major oncological conferences. Abstracts presented at plenary sessions of the American Society of Clinical Oncology (ASCO) Annual Meetings and European Society for Medical Oncology (ESMO) Congresses were collected. Trend analyses were used to analyze female contribution over time. The association between presenter's sex, study outcome (positive/negative) and journals' impact factors (IFs) of subsequently published papers was assessed using Chi‐square and Mann–Whitney *U* tests. Of 166 consecutive abstracts presented at ASCO in 2011–2018 (*n* = 34) and ESMO in 2008–2018 (*n* = 132), 21% had female presenters, all originating from Northern America (*n* = 17) or Europe (*n* = 18). The distribution of presenter's sex was similar over time (*p* = 0.70). Of 2,425 contributing authors to these presented abstracts, 28% were women. The proportion of female abstract authors increased over time (*p* < 0.05) and was higher in abstracts with female (34%) compared to male presenters (26%; *p* < 0.01). Presenter's sex was not associated with study outcome (*p* = 0.82). Median journals' IFs were lower in papers with a female first author (*p* < 0.05). In conclusion, there is a clear gender disparity in research presentations at two major oncological conferences, with 28% of authors and 21% of presenters of these studies being female. Lack of visibility of female presenters could impair acknowledgement for their research, opportunities in their academic career and even hamper heterogeneity in research.

## Abstract 2

**What's new?**

Presenting one's research at a conference is a great way to get your name and ideas heard within the professional community. In this study, the authors investigated how often women served as presenters at plenary sessions of ASCO Annual Meetings and ESMO Congresses. Looking through 166 abstracts over a period of 8 years, they found that 21% had female presenters, while 28% of study authors were female. Lack of visibility for female researchers at conferences can slow their career progress, and greater representation should be encouraged.

## Introduction

Gender inequalities in science and medicine are increasingly brought to the fore. Despite an expanding number of women entering the field of medicine, female physicians are still at disadvantage in obtaining jobs, less rewarded than men and underrepresented in leadership positions.^1^, ^2^, ^3^, ^4^, ^5^ In medical research, gender differences are even more pronounced: women are less likely to hold first‐author positions on top publications, receive requested grants, be invited as a peer reviewer, or become a full professor.^1^, ^4^, ^5^, ^6^, ^7^

Gender discrepancies in authorships of scientific publications have been identified in many disciplines all over the world, including oncology.^2^, ^8^, ^9^, ^10^, ^11^, ^12^ However, results of a clinical research project are often first brought to life through a presentation at an international conference. Such a presentation gives the scientific study an actual identity through visibility of the researcher. Presentations at major international conferences are not only important for discussion of the outcomes of a study, they also provide the presenter the opportunity for recognition for as a principal investigator, and increase the chance of climbing the academic career ladder.

Female underrepresentation in presenting studies and invitation to speak at conferences has been identified in other disciplines.^13^, ^14^, ^15^, ^16^, ^17^, ^18^ The exact share of women presenting at major oncological conferences is not clear. In our study, we aimed to identify potential gender‐based differences in contributions to presentations at two major international oncological conferences: the American Society of Clinical Oncology (ASCO) Annual Meetings and European Society for Medical Oncology (ESMO) Congresses.

## Methods

### Data collection

We aimed to collect consecutive abstracts of all plenary sessions of ASCO Annual Meetings and presidential sessions of ESMO Congresses between 2000 and 2018. The abstracts presented at these sessions are assumed to have the highest impact on oncological research and practice. Specific data on ASCO abstracts were available from 2011 and on ESMO abstracts from 2008.

Data on ASCO abstracts, including sexes of the presenters, were provided by ASCO Center for Research and Analytics for all abstracts presented at the plenary sessions since 2011. All consecutive ESMO abstracts presented at the presidential sessions since 2008 were identified from the ESMO website (http://www.esmo.org) or the website of the conference. Data extracted from the abstracts included information on presenters, names and order of authors, country of origin, study subject and results. Sexes of presenters and authors were interpreted based on their first names or, if inconclusive, based on available online information including photos and electronic portfolio of the specific author. Study results were defined as positive and negative if they met or did not meet the primary endpoints, respectively, and neither negative nor positive if results were not clear yet, or if both positive and negative results were found.

From all abstracts, the subsequently published papers were identified and corresponding impact factors (IFs) of the journals in which they were published (obtained from InCites Journal Citation Reports) were collected. One‐year IFs of the year in which the article was published were used, or of the previous year in case IFs were not yet known. Any changes in authorships compared to the presented abstract were identified.

Ethical approval to perform our study was not considered to be necessary.

### Statistical analysis

Descriptive statistics were used to display the distribution of presenter's and abstract author's sex. Chi‐square or Fisher's exact tests where appropriate were used to compare the sex distribution in abstract presenters and authors per year. The association between presenter's or last author's sex and distribution of author's sex, study outcome and IFs were analyzed using Chi‐square and Mann–Whitney *U* tests, respectively. A trend in contribution of both sexes in presenters and abstract authors over time was tested using the Cochran‐Armitage trend test; *p*‐values lower than 0.05 were regarded as statistically significant. Statistical analyses were performed using SAS software (version 9.4, SAS institute, Cary, NC).

### Data availability

The data that support the findings of our study are available from the corresponding author upon reasonable request.

## Results

### Presenters

Data of 166 consecutive abstracts presented at plenary sessions of ASCO Annual Meetings from 2011 and at ESMO Congresses from 2008 were collected. Included abstracts of the plenary sessions of ASCO Annual Meetings between 2011 and 2018 (*n* = 34) and of the presidential sessions of ESMO conferences between 2008 and 2018 (*n* = 132) are shown in Tables 1 and 2, respectively. References of all of these abstracts and subsequently published papers can be found in the Supplementary Material.

**Table 1:** Abstracts presented at ASCO annual meetings

		Presenter			Abstract							Article					
Year	Abstract no.	Name	Sex	Country of origin	Author place presenter	Sex last author	No. of authors	No. of male authors	No. of female authors	No. of authors unknown sex	Study outcome^1^	Journal published	Year	IF	Sex of the first author	Sex of the last author	Subject
2011	A‐2011‐1^41^	H. Joensuu	M	Finland	First	M	18	13	5	0	P	JAMA J Am Med Assoc^42^	2012	29.978	M	M	GIST
	A‐2011‐2^43^	R.L. Ladenstein	F	Austria	First	F	19	9	10	0	P	Lancet Oncol^44^	2017	36.418	F	F	Neuroblastoma
	A‐2011‐3^45^	E.C. Larsen	M	United States	First	M	16	8	8	0	P	J Clin Oncol^46^	2016	24.008	M	M	Leukemia
	A‐2011‐4^47^	P.B. Chapman	M	United States	First	M	20	17	3	0	P	New Engl J Med^48^	2011	53.298	M	M	Melanoma
	A‐2011‐5^49^	J.D. Wolchok	M	United States	First	F	10	9	1	0	P	New Engl J Med^50^	2011	53.298	F	M	Melanoma
2012	A‐2012‐1^51^	K.L. Blackwell	F	United States	First	M	14	10	4	0	P	New Engl J Med^52^	2012	51.658	M	F	Breast cancer
	A‐2012‐2^53^	M.J. Van Den Bent	M	The Netherlands	First	M	19	15	4	0	P	J Clin Oncol^54^	2013	17.879	M	M	Oligodendroglioma
	A‐2012‐3^55^	M.J. Rummel	M	Germany	First	M	18	15	3	0	P	Lancet^56^	2013	39.207	M	M	Lymphoma
	A‐2012‐4^57^	M. Hussain	F	United States	First	M	18	13	5	0	N	New Engl J Med^58^	2013	54.420	F	M	Prostate cancer
2013	A‐2013‐1^59^	M.R. Gilbert	M	United States	First	M	20	15	5	0	N	New Engl J Med^60^	2014	55.873	M	M	Glioblastoma
	A‐2013‐2^61^	S.S. Shastri	M	India	First	M	6	4	2	0	P	JNCI J Natl Cancer I^62^	2014	12.583	M	M	Cervical cancer
	A‐2013‐3^63^	K.S. Tewari	M	United States	First	M	10	6	4	0	P	New Engl J Med^64^	2014	55.873	M	M	Cervical cancer
	A‐2013‐4^65^	M.S. Brose	F	United States	First	M	16	12	4	0	P	Lancet^66^	2014	45.217	F	M	Thyroid cancer
	A‐2013‐5^67^	R.G. Gray	M	United kingdom	First	M	22	15	7	0	P	Not (yet) published					Breast cancer
2014	A‐2014‐1^68^	O. Pagani	F	Switzerland	First	F	20	10	10	0	P	New Engl J Med^69^	2014	55.873	F	F	Breast cancer
	A‐2014‐2^70^	C. Sweeney	M	United States	First	M	17	15	2	0	P	New Engl J Med^71^	2015	59.558	M	M	Prostate cancer
	A‐2014‐3^72^	A.P. Venook	M	United States	First	M	15	11	4	0	N	JAMA J Am Med Assoc^73^	2017	47.661	M	M	Colorectal cancer
	A‐2014‐4^74^	M.J. Piccart	F	Belgium	First	F	20	15	5	0	N/P	Not (yet) published					Breast cancer
2015	A‐2015‐1^75^	J.D. Wolchok	M	United States	First	M	20	17	3	0	P	New Engl J Med^76^	2015	59.558	M	M	Melanoma
	A‐2015‐2^77^	G.T. Armstrong	M	United States	First	M	15	9	6	0	P	New Engl J Med^78^	2016	72.406	M	M	Childhood cancers
	A‐2015‐3^79^	A. D'Cruz	M	India	First	M	16	6	10	0	P	New Engl J Med^80^	2015	59.558	M	M	Oral cancer
	A‐2015‐4^81^	P.D. Brown	M	United States	First	M	17	10	7	0	N	JAMA J Am Med Assoc^82^	2016	44.405	M	M	Multiple types of cancer
2016	A‐2016‐1^83^	P.E. Goss	M	United States	First	F	20	11	9	0	P	New Engl J Med^84^	2016	72.406	M	F	Breast cancer
	A‐2016‐2^85^	J.R. Perry	M	Canada	First	M	20	16	4	0	P	New Engl J Med^86^	2017	79.260	M	M	Glioblastoma
	A‐2016‐3^87^	J.R. Park	F	United States	First	F	17	7	10	0	P	Not (yet) published					Neuroblastoma
	A‐2016‐4^88^	A. Palumbo	M	Italy	First	M	19	13	5	1	P	New Engl J Med^89^	2016	72.406	M	M	Multiple myeloma
2017	A‐2017‐1^90^	Q. Shi	F	United States	First	M	20	16	4	0	N/P	New Engl J Med^91^	2018	70.670	M	M	Colorectal cancer
	A‐2017‐2^92^	E.M. Basch	M	United States	First	F	13	6	7	0	P	JAMA J Am Med Assoc^93^	2017	47.661	M	F	Multiple types of cancer
	A‐2017‐3^94^	K. Fizazi	M	France	First	M	15	11	3	1	P	New Engl J Med^95^	2017	79.260	M	M	Prostate cancer
	A‐2017‐4^96^	M.E. Robson	M	United States	First	M	14	6	8	0	P	New Engl J Med^97^	2017	79.260	M	M	Breast cancer
2018	A‐2018‐1^98^	J.A. Sparano	M	United States	First	M	20	14	6	0	P	New Engl J Med^99^	2018	70.670	M	M	Breast cancer
	A‐2018‐2^100^	G. Bisogno	M	Italy	First	M	12	6	6	0	P	Lancet Oncol^101^	2018	35.386	M	M	Rhabdomyosarcoma
	A‐2018‐3^102^	A. Mejean	M	France	First	M	20	18	2	0	P	New Engl J Med^103^	2018	70.670	M	M	Renal cell carcinoma
	A‐2018‐4^104^	G. Lopes	M	United States	First	M	13	10	2	1	P	Lancet^105^	2019	59.102	M	M	Lung cancer
Total	*N* = 34		F: *N* = 8			F: *N* = 7	569	388	178	3		*N* = 31			F: *N* = 5	F: *N* = 5	

1 Abstracts presented at plenary sessions of ASCO annual meetings between 2011 and 2018. For papers published in 2019, journal IFs of 2018 were used.

Abbreviations: ASCO, American Society of Clinical Oncology; F, female; GIST, gastrointestinal stroma cell tumor; IF, impact factor; M, male; N, negative; N/P, outcome did not reach significance or endpoint, but did show improvement/benefit or reached some of the outcomes; no., number; P, positive.

**Table 2:** abstracts presented at ESMO congresses

		Presenter			Abstract							Article					
Year	Abstract no.	Name	Sex	Country of origin	Author place presenter	Sex of the last author	No. of authors	No. of male authors	No. of female authors	No. of authors unknown sex	Study outcome^1^	Journal published	Year	IF	Sex of the first author	Sex of the last author	Subject
2008	E‐2008‐1^106^	C. Manegold	M	Germany	First	M	10	6	4	0	P	J Clin Oncol^107^	2009	17.793	M	M	Lung cancer
	E‐2008‐2^108^	T. Mok	M	Hong Kong	First	M	10	6	4	0	P	New Engl J Med^109^	2009	47.050	M	M	Lung cancer
	E‐2008‐3^110^	R.S.J. Midgley	F	United Kingdom	First	M	10	5	5	0	N	J Clin Oncol^111^	2010	18.970	F	M	Colorectal cancer
	E‐2008‐4^112^	B.J. Monk	M	United States	First	M	10	8	2	0	P	J Clin Oncol^113^	2010	18.970	M	F	Ovarian cancer
	E‐2008‐5^114^	S. Lee	M	United Kingdom	First	F	5	1	4	0	N	J Clin Oncol^115^	2010	18.970	M	M	Glioma
	E‐2008‐6^116^	C. Karapetis	M	Australia	First	M	10	7	3	0	P	New Engl J Med^117^	2008	50.017	M	M	Colorectal cancer
	E‐2008‐7^118^	M. Löhr	M	Germany	First	M	10	9	1	0	P	Ann Oncol^119^	2012	7.384	M	M	Pancreatic cancer
	E‐2008‐8^120^	P.M. Patel	M	United Kingdom	First	M	10	6	4	0	N	Eur J Cancer^121^	2011	5.536	M	M	Melanoma
	E‐2008‐9^122^	M. Auerbach	M	United States	First	M	8	6	2	0	P	Am J Hematol^123^	2010	3.576	M	M	Multiple types of cancer
2009	E‐2009‐1^124^	M. van Hemelrijck	F	United Kingdom	First	M	8	6	2	0	P	J Clin Oncol^125^	2010	18.970	F	M	Prostate cancer
	E‐2009‐2^126^	C. van de Velde	M	The Netherlands	First	M	10	8	2	0	P	Lancet^127^	2011	38.278	M	M	Breast cancer
	E‐2009‐3^128^	A. M. Brunt	M	United Kingdom	First	M	10	6	4	0	P	Radiother Oncol^129^	2011	5.580	N/A	N/A	Breast cancer
	E‐2009‐4^130^	R. Issels	M	Germany	First	M	10	10	0	0	P	Lancet Oncol^131^	2010	17.764	M	M	Soft‐tissue sarcoma
	E‐2009‐5^132^	A. Stopeck	F	United States	First	F	10	5	5	0	P	J Clin Oncol^133^	2010	18.970	M	F	Breast cancer
	E‐2009‐6^134^	M.E.L. van der Burg	F	The Netherlands	First	M	2	1	1	0	N	Lancet^135^	2010	33.633	M	F	Ovarian cancer
	E‐2009‐7^136^	G.G. Steger	M	Germany	First	M	10	8	2	0	P	Ann Oncol^137^	2014	7.040	M	M	Breast cancer
	E‐2009‐8^138^	J. Baselga	M	Spain	First	M	10	8	2	0	P	J Clin Oncol^139^	2012	18.038	M	M	Breast cancer
	E‐2009‐9^140^	M. Baumann	M	Germany	First	M	10	8	2	0	N/P	Radiother Oncol^141^	2011	5.580	M	M	Lung cancer
	E‐2009‐10^142^	D. Hailer	M	United States	First	M	10	9	1	0	P	J Clin Oncol^143^	2015	20.982	M	M	Colorectal cancer
	E‐2009‐11^144^	T. Maughan	M	United Kingdom	First	M	10	9	1	0	N	Lancet^145^	2011	38.278	M	M	Colorectal cancer
	E‐2009‐12^146^	S. Badve	M	United States	First	M	10	7	3	0	P	Not (yet) published					Breast cancer
	E‐2009‐13^147^	P. Chapman	M	United States	First	M	10	10	0	0	P	New Engl J Med^148^	2010	53.486	M	M	Melanoma
	E‐2009‐14^149^	B. Johnson	M	United States	First	M	7	6	1	0	P	J Clin Oncol^150^	2013	17.879	M	M	Lung cancer
	E‐2009‐15^151^	A. Inoue	M	Japan	First	M	10	10	0	0	P	Ann Oncol^152^	2013	6.578	M	M	Lung cancer
	E‐2009‐16^153^	J. Douillard	M	France	First	F	10	9	1	0	P	J Clin Oncol^154^	2010	18.970	M	F	Colorectal cancer
	E‐2009‐17^155^	C. Osborne	F	United States	Second	M	10	6	4	0	P	New Engl J Med^156^	2011	53.298	F	M	Breast cancer
	E‐2009‐18^157^	A. Dueñas‐González	M	Mexico	First	M	11	8	3	0	P	J Clin Oncol^158^	2011	18.372	M	M	Cervical cancer
	E‐2009‐19^159^	E. van Cutsem	M	Belgium	First	M	10	7	3	0	P	Lancet^160^	2010	33.633	M	M	Gastric cancer
	E‐2009‐20^161^	C. Nutting	M	United Kingdom	First	F	10	8	2	0	P	Lancet Oncol^162^	2011	22.589	M	F	Head and neck cancer
	E‐2009‐21^163^	A.M.M. Eggermont	M	The Netherlands	First	M	5	4	1	0	P	Eur J Cancer^164^	2012	5.061	M	M	Melanoma
	E‐2009‐22^165^	E.L. Kwak	F	United States	First	M	10	9	1	0	P	Not (yet) published					Multiple types of cancer
2010	E‐2010‐1^166^	V.A. Miller	M	United States	First	M	10	8	2	0	N/P	Lancet Oncol^167^	2012	25.117	M	M	Lung cancer
	E‐2010‐2^168^	J. Chih‐Hsin Yang	M	Taiwan	First	M	10	7	3	0	N	J Clin Oncol^169^	2011	18.372	M	M	Lung cancer
	E‐2010‐3^170^	E.A. Perez	F	United States	First	F	10	5	5	0	P	Breast Cancer Res^171^	2014	5.490	F	M	Breast cancer
	E‐2010‐4^172^	T.J. Perren	M	United Kingdom	First	M	10	9	1	0	P	New Engl J Med^173^	2011	53.298	M	M	Ovarian cancer
	E‐2010‐5^174^	J.S. De Bono	M	United Kingdom	First	M	10	10	0	0	P	New Engl J Med^175^	2011	53.298	M	M	Prostate cancer
2011	E‐2011‐1^176^	L. Dirix	M	Belgium	First	M	9	7	1	1	P	New Engl J Med^177^	2012	51.658	M	M	Basal cell carcinoma
	E‐2011‐2^178^	C. Parker	M	United Kingdom	First	M	10	9	1	0	P	New Engl J Med^179^	2013	54.420	M	M	Prostate cancer
	E‐2011‐3^180^	J. Bourhis	M	Switzerland	First	F	17	15	2	0	N	Lancet Oncol^181^	2012	25.117	M	F	Head and neck cancer
	E‐2011‐4^182^	M. Bebin	F	United Kigdom	First	M	10	7	3	0	P	Lancet^183^	2013	39.207	M	M	Astrocytoma
	E‐2011‐5^184^	I. Fernando	M	United Kingdom	First	M	10	8	2	0	P	Not (yet) published					Breast cancer
	E‐2011‐6^185^	J. Tabernero	M	Spain	First	F	12	9	3	0	P	Eur J Cancer^186^	2014	5.417	M	F	Colorectal cancer
	E‐2011‐7^187^	C. Aghajanian	F	United States	First	F	9	2	7	0	P	J Clin Oncol^188^	2012	18.038	F	M	Ovarian cancer
	E‐2011‐8^189^	P. Hoskin	M	United Kingdom	First	M	13	9	4	0	N	JNCI J Natl Cancer I^190^	2015	11.370	M	M	Prostate cancer
	E‐2011‐9^191^	R. Sullivan	M	United Kingdom	First	M	10	10	0	0	N/A	Lancet Oncol^192^	2011	22.589	M	M	Multiple types of cancer
	E‐2011‐10^193^	L. Krug	M	United States	First	M	10	9	1	0	N	Lancet Oncol^194^	2015	26.509	M	M	Mesothelioma
	E‐2011‐11^195^	J. Baselga	M	United States	First	M	10	8	2	0	P	Ann Oncol^196^	2014	7.040	F	M	Breast cancer
	E‐2011‐12^197^	E.J.T. Rutgers	M	The Netherlands	Last	M (= presenter)	16	9	7	0	P	Eur J Cancer^198^	2011	5.536	M	F	Breast cancer
	E‐2011‐13^199^	H.J. Bonjer	M	The Netherlands	First	M	7	6	1	0	P	New Engl J Med^200^	2015	59.558	M	F	Colorectal cancer
	E‐2011‐14^201^	M. Van Hemelrijck	F	United Kingdom	First	M	7	4	3	0	P	Hypertension^202^	2012	6.873	F	F	Multiple types of cancer
	E‐2011‐15^203^	F. Amant	M	Belgium	First	F	16	9	7	0	N/P	Lancet Oncol^204^	2012	25.117	M	F	Multiple types of cancer
	E‐2011‐16^205^	E. Papaemmanuil	F	United Kingdom	First	M	10	7	3	0	P	New Engl J Med^206^	2011	53.298	F	M	Myelodysplastic malignancies
	E‐2011‐17^207^	M. Middleton	M	United Kingdom	First	M	10	9	1	0	N/P	Ann Oncol^208^	2015	9.269	M	M	Melanoma
	E‐2011‐18^209^	E. van Cutsem	M	Belgium	First	M	11	9	2	0	P	Ann Oncol^210^	2015	9.269	M	M	Colorectal cancer
2012	E‐2012‐1^211^	A. Shaw	F	United States	First	M	20	14	6	0	P	New Engl J Med^212^	2013	54.420	F	M	Lung cancer
	E‐2012‐2^213^	A.X. Zhu	M	United States	First	M	14	13	1	0	N	J Clin Oncol^214^	2015	20.982	M	M	Hepatocellular carcinoma
	E‐2012‐3^215^	F. Lordick	M	Germany	First	M	16	12	4	0	N	Lancet Oncol^216^	2013	24.725	M	M	Gastric cancer
	E‐2012‐4^217^	J. Taieb	M	France	First	M	19	16	3	0	N	Lancet Oncol^218^	2014	24.690	M	M	Colorectal cancer
	E‐2012‐5^219^	X. Pivot	M	France	First	M	19	14	5	0	N	Lancet Oncol^220^	2013	24.725	M	M	Breast cancer
	E‐2012‐6^221^	R. Gelber	M	United States	Second	M	24	19	5	0	N	Lancet^222^	2013	39.207	M	M	Breast cancer
	E‐2012‐7^223^	W. Van der Graaf	F	The Netherlands	Last	F (= presenter)	19	15	4	0	N	Lancet Oncol^224^	2014	24.690	M	F	Soft‐tissue sarcoma
	E‐2012‐8^225^	R.J. Motzer	M	United States	First	M	25	18	7	0	P	New Engl J Med^226^	2013	54.420	M	M	Renal cell carcinoma
2013	E‐2013‐1^227^	P. Autier	M	France	First	M	4	3	1	0	N	Lancet Diabetes Endocrinol^228^	2014	9.185	M	M	Multiple types of cancer
	E‐2013‐2^229^	P. Poortmans	M	The Netherlands	First	M	10	7	3	0	P	New Engl J Med^230^	2015	59.558	M	M	Breast cancer
	E‐2013‐3^231^	A.J. Breugom	F	The Netherlands	First	M	11	7	4	0	N	Lancet Oncol^232^	2015	26.509	F	M	Colorectal cancer
	E‐2013‐4^233^	M. Reimers	F	The Netherlands	First	M	10	7	3	0	P	JNCI J Natl Cancer ^234^	2014	12.583	F	M	Colorectal cancer
	E‐2013‐5^235^	G. Giaccone	M	United States	First	M	10	7	3	0	N/P	Eur J Cancer^236^	2015	6.163	M	M	Lung cancer
	E‐2013‐6^237^	P. Ruszniewski	M	France	Second	F	13	7	6	0	P	New Engl J Med^238^	2014	55.873	F	M	Neuroendocrine tumors
	E‐2013‐7^239^	P. Brastianos	F	United States	First	M	10	8	2	0	P	Cancer Discov^240^	2015	19.783	F	M	Multiple types of cancer
	E‐2013‐8^241^	P. Witteveen	F	The Netherlands	First	M	10	7	3	0	N	J Clin Oncol^242^	2014	18.428	M	F	Ovarian cancer
	E‐2013‐9^243^	A. Oza	M	Canada	First	M	13	10	3	0	N/P	Lancet Oncol^244^	2015	26.509	M	M	Ovarian cancer
	E‐2013‐10^245^	F. Sclafani	M	United Kingdom	First	M	10	7	3	0	P	Eur J Cancer^246^	2014	5.417	M	M	Colorectal cancer
	E‐2013‐11^247^	J.C. Soria	M	France	Last	M (= presenter)	17	12	5	0	N/A	Eur J Cancer^248^	2014	5.417	F	M	Multiple types of cancer
	E‐2013‐12^249^	R.E. Coleman	M	United Kingdom	First	F	10	7	3	0	N/P	Lancet Oncol^250^	2014	24.690	M	F	Breast cancer
	E‐2013‐13^251^	J. Ledermann	M	United Kingdom	First	M	10	7	3	0	P	Lancet^252^	2016	47.831	M	M	Ovarian cancer
	E‐2013‐14^253^	P. Van Loo	M	United Kingdom	Last	M (= presenter)	10	7	3	0	P	Nat Commun^254^	2017	12.353	F	M	Multiple types of cancer
	E‐2013‐15^255^	J.G. Eriksen	M	Denmark	First	M	10	8	2	0	N	Not (yet) published					Head and neck cancer
	E‐2013‐16^256^	R. Chlebowski	M	United States	First	F	11	8	3	0	P	JNCI J Natl Cancer I^257^	2016	12.589	M	F	Endometrial cancer
	E‐2013‐17^258^	H.J. de Koning	M	The Netherlands	First	F	9	7	2	0	N	Ann Intern Med^259^	2014	17.810	M	F	Lung cancer
2014	E‐2014‐1^260^	J.S. Weber	M	United States	First	M	20	17	3	0	P	Lancet Oncol^261^	2015	26.509	M	M	Melanoma
	E‐2014‐2^262^	C. Robert	F	France	First	M	20	14	6	0	P	Lancet Oncol^263^	2015	26.509	M	F	Melanoma
	E‐2014‐3^264^	G.A. McArthur	M	Australia	First	F	17	12	5	0	P	Lancet Oncol^265^	2016	33.900	M	M	Melanoma
	E‐2014‐4^266^	S. Swain	F	United States	First	M	14	9	5	0	P	New Engl J Med^267^	2015	59.558	F	M	Breast cancer
	E‐2014‐5^268^	J.F. Vansteenkiste	M	Belgium	First	M	20	19	1	0	N	Lancet Oncol^269^	2016	33.900	M	M	Lung cancer
	E‐2014‐6^270^	T.S. Mok	M	Hong Kong	First	M	18	14	4	0	N	J Clin Oncol^271^	2017	26.303	M	M	Lung cancer
2015	E‐2015‐1^272^	M. Sant	F	Italy	First	F	18	8	10	0	P	Eur J Cancer^273^	2015	6.163	F	M	Multiple types of cancer
	E‐2015‐2^274^	R. Atun	M	United States	First	F	18	12	6	0	P	Lancet Oncol^275^	2015	26.509	M	F	Multiple types of cancer
	E‐2015‐3^276^	P. Sharma	F	United States	First	M	15	12	3	0	P	Eur Urol^277^	2017	17.581	M	M	Renal cell carcinoma
	E‐2015‐4^278^	T. Choueiri	M	United States	First	M	23	17	6	0	P	New Engl J Med^279^	2015	59.558	M	M	Renal cell carcinoma
	E‐2015‐5^280^	C. Vrieling	F	Switzerland	First	M	11	8	3	0	P	JAMA Oncol^281^	2017	20.871	F	M	Breast cancer
	E‐2015‐6^282^	J. Yao	M	United States	First	F	22	18	4	0	P	Lancet^283^	2016	47.831	M	F	Neuroendocrine tumors
	E‐2015‐7^284^	P. Ruszniewski	M	France	Second last	M	14	12	2	0	P	New Engl J Med^285^	2017	79.260	M	M	Neuroendocrine tumors
	E‐2015‐8^286^	C. Oude Ophuis	F	The Netherlands	First	M	11	8	3	0	N	Eur J Surg Oncol^287^	2016	3.522	F	M	Melanoma
	E‐2015‐9^288^	R.A. Stahel	M	Switzerland	First	M	20	15	5	0	P	Lancet Respir Med^289^	2017	21.466	M	M	Lung cancer
	E‐2015‐10^290^	M.C. Pietanza	F	United States	First	M	15	12	3	0	P	Lancet Oncol^291^	2017	36.418	M	M	Lung cancer
	E‐2015‐11^292^	D. Dearnaley	M	United Kingdom	First	F	20	10	10	0	N/P	Lancet Oncol^293^	2016	33.900	M	F	Prostate cancer
	E‐2015‐12^294^	R. Sullivan	M	United Kingdom	First	M	43	37	6	0	N/A	Lancet Oncol^295^	2015	26.509	M	M	Multiple types of cancer
	E‐2015‐13^296^	M. Carducci	M	United States	First	F	19	16	3	0	P	J Clin Oncol^297^	2016	24.008	F	M	Prostate cancer
	E‐2015‐14^298^	J. Sparano	M	United States	First	M	20	11	9	0	P	New Engl J Med^99^	2018	70.670	M	M	Breast cancer
2016	E‐2016‐1^299^	G.N. Hortobagyi	M	United States	First	F	20	13	7	0	P	New Engl J Med^300^	2016	72.406	M	F	Breast cancer
	E‐2016‐2^301^	A.M. Eggermont	M	France	First	M	19	13	6	0	P	New Engl J Med^302^	2016	72.406	M	M	Melanoma
	E‐2016‐3^303^	M. Mirza	M	Denmark	First	F	20	14	6	0	P	New Engl J Med^304^	2016	72.406	M	F	Ovarian cancer
	E‐2016‐4^305^	K. Harrington	M	United Kingdom	First	M	11	6	5	0	P	Lancet Oncol^306^	2017	36.418	M	F	Head and neck cancer
	E‐2016‐5^307^	C. Langer	M	United States	First	F	19	13	6	0	P	Lancet Oncol^308^	2016	33.900	M	M	Lung cancer
	E‐2016‐6^309^	M. Reck	M	Germany	First	F	18	9	9	0	P	New Engl J Med^310^	2016	72.406	M	F	Lung cancer
	E‐2016‐7^311^	M. Socinski	M	United States	First	M	20	14	6	0	N	New Engl J Med^312^	2017	79.260	M	M	Lung cancer
	E‐2016‐8^313^	F. Barlesi	M	France	First	M	20	18	2	0	P	Lancet^314^	2017	53.254	M	M	Lung cancer
	E‐2016‐9^315^	A. Gronchi	M	Italy	First	M	19	15	4	0	P	Lancet Oncol^316^	2017	36.418	M	M	Soft‐tissue sarcoma
	E‐2016‐10^315^	K. Fizazi	M	France	First	M	13	9	4	0	N	Lancet Oncol^317^	2017	36.418	M	M	Prostate cancer
	E‐2016‐11^318^	T.K. Choueiri	M	United States	First	M	12	10	2	0	P	J Clin Oncol^319^	2017	26.303	M	M	Renal cell carcinoma
	E‐2016‐12^320^	A. Ravaud	M	France	First	M	20	16	3	1	P	New Engl J Med^321^	2016	72.406	M	M	Renal cell carcinoma
2017	E‐2017‐1^322^	L. Paz‐Ares	M	Spain	First	M	20	17	3	0	P	New Engl J Med^323^	2017	79.260	M	M	Lung cancer
	E‐2017‐2^324^	V. Westeel	F	France	First	M	20	17	3	0	N	Not (yet) published					Lung cancer
	E‐2017‐3^325^	S. Ramalingam	M	United States	First	M	18	12	6	0	P	New Engl J Med^326^	2018	70.670	M	M	Lung cancer
	E‐2017‐4^327^	A. Di Leo	M	Italy	First	M	17	10	7	0	P	J Clin Oncol^328^	2017	26.303	M	M	Breast cancer
	E‐2017‐5^329^	S. Gupta	M	India	First	M	20	8	12	0	N	J Clin Oncol^330^	2018	26.303	M	M	Cervical cancer
	E‐2017‐6^331^	D. Petrylak	M	United States	First	M	20	14	6	0	P	Lancet^332^	2017	53.254	M	F	Renal cell carcinoma
	E‐2017‐7^333^	B. Escudier	M	France	First	M	20	15	5	0	P	New Engl J Med^334^	2018	70.670	M	M	Renal cell carcinoma
	E‐2017‐8^335^	K. Lewis	M	United States	First	M	14	13	0	1	N/P	Lancet Oncol^336^	2018	36.418	M	M	Melanoma
	E‐2017‐9^337^	A. Hauschild	M	Germany	First	M	19	12	7	0	P	New Engl J Med^338^	2017	79.260	F	M	Melanoma
	E‐2017‐10^339^	J. Weber	M	United States	First	M	20	12	8	0	P	New Engl J Med^334^	2017	79.260	M	M	Melanoma
2018	E‐2018‐1^340^	P. Schmid	M	United Kingdom	First	F	18	7	11	0	P	New Engl J Med^341^	2018	70.670	M	F	Breast cancer
	E‐2018‐2^342^	M. Cristofanilli	M	United States	First	M	19	9	10	0	P	New Engl J Med^343^	2018	70.670	M	M	Breast cancer
	E‐2018‐3^344^	F. André	M	France	First	M	20	11	8	1	P	New Engl J Med^345^	2019	70.670	M	M	Breast cancer
	E‐2018‐4^346^	Z. Jiang	M	China	First	M	19	11	4	4	P	Lancet Oncol^347^	2019	35.386	M	M	Breast cancer
	E‐2018‐5^348^	A. Hoyle	M	United Kingdom	First	M	20	18	2	0	P	Not (yet) published					Prostate cancer
	E‐2018‐6^349^	C. Parker	M	United Kingdom	First	M	19	15	4	0	N	Lancet^350^	2018	59.102	M	M	Prostate cancer
	E‐2018‐7^351^	R. Motzer	M	United States	First	M	20	16	3	1	P	New Engl J Med^352^	2019	70.670	M	M	Renal cell carcinoma
	E‐2018‐8^353^	K. Moore	F	United States	First	M	19	10	9	0	P	New Engl J Med^354^	2018	70.670	F	M	Ovarian cancer
	E‐2018‐9^355^	B. Burtness	F	United States	First	M	20	12	7	1	P	Not (yet) published					Head and neck cancer
	E‐2018‐10^356^	H. Mehanna	M	United Kingdom	First	F	20	14	6	0	N	Lancet^357^	2019	59.102	M	F	Oropharyngeal cancer
	E‐2018‐11^358^	C. Zhou	M	China	First	M	18	8	4	6	P	Lancet Respir Med^359^	2019	22.992	M	M	Lung cancer
Total	*N* = 132		F: *N* = 27			F: *N* = 26	1,856	1,340	500	16	P	*N* = 125			F: *N* = 23	F: *N* = 27	

1 Abstracts presented at presidential symposia of ESMO Congresses (2006, 2008, 2010, 2012, 2014, 2006–2018), and ESMO/ECCO conferences (2009, 2013, 2015). Presenters were last abstract authors in E‐2011‐12, E‐2012‐7, E‐2013‐11, and E‐2013‐14, and therefore, presenter's and last abstract author's sex are similar. For papers published in 2019, journal impact factors of 2018 were used.

Abbreviations: ECCO, European Cancer Organization; ESMO, European Society for Medical Oncology; F, female; IF, impact factor; M, male; N, negative; N/A, not applicable; no., number; N/P, outcome did not reach significance or endpoint, but did show improvement/benefit or reached some of the outcomes; P, positive.

**Figure 1:**
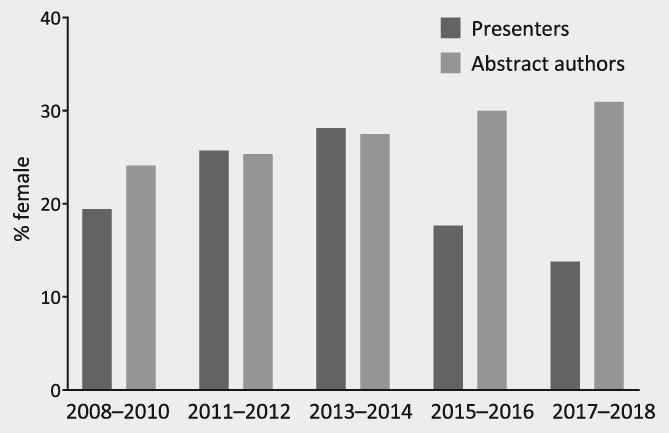
Proportion of female presenters and abstract authors over time at plenary sessions of American Society of Clinical Oncology (ASCO) Annual Meetings and European Society for Medical Oncology (ESMO) Congresses. Results of 2008–2010 is based on ESMO abstracts solely. Abstract authors with unknown sex (*n* = 19) are not displayed.

Of all 166 abstracts, 35 (21%) were presented by a woman. Although the proportion of female presenters has decreased since 2015–2016 (Fig. 1), the distribution of female and male contribution to presenters was not different over the years (*p* = 0.699), neither was a trend observed in contribution of both sexes over time (*p* = 0.350).

The majority of the presenters originated from Europe (*n* = 90, 54%), followed by Northern America (*n* = 65, 39%), Asia (*n* = 9, 5%) and Oceania (*n* = 2, 1%). All female presenters came from Northern America (*n* = 17) or Europe (*n* = 18). The share of women of all Northern American and European presenters was 26 and 20%, respectively. Per country, 17 of 62 (27%) American, 5 of 29 (17%) British, 1 of 6 (17%) Belgian, 2 of 17 (12%) French, 6 of 13 (46%) Dutch, 2 of 4 (50%) Swiss, 1 of 5 (20%) Italian presenters and the only Austrian presenter were female.

Almost a quarter of the studies presented by a female researcher (*n* = 35) concerned breast cancer (*n* = 8, 23%), lung cancer (*n* = 3, 9%), followed by ovarian cancer, colorectal cancer and multiple types of cancer (all: *n* = 4, 11%). Other subjects are shown in Tables 1 and 2. Overall, 26% of the presentations about breast cancer, 44% about ovarian cancer, 29% about colorectal cancer and 17% about lung cancer were presented by a woman.

Study outcomes were most often positive (*n* = 119, 71%), while 33 (20%) had negative outcomes and 14 (8%) neither positive nor negative (N/P), or nonapplicable (N/A). Outcomes were positive, negative and N/P or N/A in 71, 23 and 6% of the 35 studies presented by a female researcher, and 72, 19 and 9% of 131 abstracts with male presenters, respectively. The outcomes of presented abstracts did not differ between male and female presenters (*p* = 0.746). Presenter's sex was not associated with study outcome (*p* = 0.815).

### Abstract authors

Figure 1 shows the overall proportion of female presenters and abstract authors. Of all authors of the presented abstracts (*n* = 2,425), 679 (28%) were female, 1,728 (71%) were male and sex was unknown in 19 (1%) authors. The distribution of sex of abstract authors differed statistically significantly over the years (*p* = 0.046), and a positive trend was observed in contribution of female authors over time (*p* = 0.007). The number of female authors was higher in abstracts with a female presenter (34%) compared to abstracts with a male presenter (26%; *p* = 0.001).

Overall, contribution of women to last abstract authorship was 20% (*n* = 33). Last abstracts' authors were female in 9/35 (26%) of the studies presented by a woman and in 23/131 (18%) of studies presented by a male researcher (*p* = 0.277).

Sex of the last abstract author was not associated with study outcomes (*p* = 0.433).

### Subsequently published papers

The majority of the 166 presented abstracts were subsequently published in an international journal (*n* = 156, 94%). In 56 (36%) of these 156 papers, either the first or last author was a woman. Female researchers were involved as first author in 29 (19%) and last author in 32 (21%) articles.

A total of 30/35 (86%) abstracts presented by a woman were published as article, which was statistically significantly less than the 126/131 (96%) abstracts with a male presenter that resulted in a paper (*p* = 0.021). In 4/30 (13%) articles, the female presenter of the abstract was not involved as first, second or last author, and the first authors of these papers were all males (A‐2017‐1, E‐2011‐4, E‐2013‐8 and E‐2015‐10; Tables 1 and 2). In 3/126 (2%) published papers with a male abstract presenter, the presenter was not first, second or last author of the article, and all the first authors were other males (E‐2010‐2, E‐2011‐1, E‐2017‐1; Table 2).

Median IF of journals of papers with a female first author was 20.3 (interquartile range [IQR], 8.4, 53.4), which was lower than of papers with a male first author (median IF 35.4 [IQR, 20.5, 59.1]; *p* = 0.046). Sex of the presenter, last abstract author, or last author of the manuscript were not associated with IF of journals of subsequently published papers (*p* = 0.101, *p* = 0.864 and *p* = 0.922, respectively).

### ASCO *vs*. ESMO

**Figure 2:**
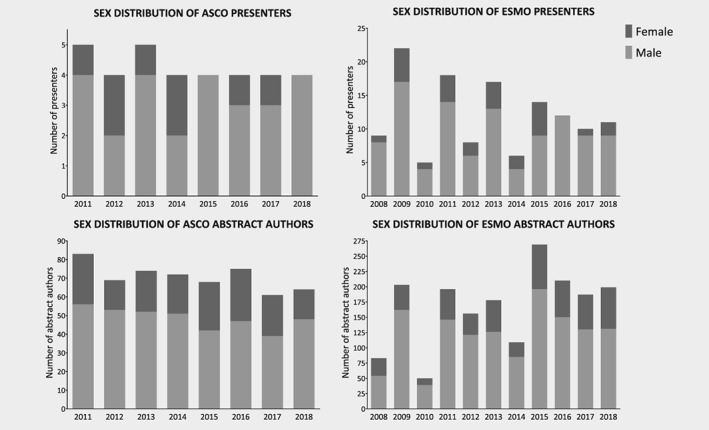
Distribution of sex in both American Society of Clinical Oncology (ASCO) and European Society for Medical Oncology (ESMO) abstract presenters and authors.

Figure 2 shows the sex distribution of abstract presenters in both ASCO and ESMO conferences. The distribution of sex of presenters did not differ between ASCO and ESMO (*p* = 0.756), but the proportion of female authors in ASCO abstracts (32%) was significantly higher compared to those of ESMO (27%; *p* = 0.048).

When analyzing the meetings separately, we found a statistically significant positive trend in female contribution observed in ESMO abstract authors (*p* = 0.014), which was not found in ASCO abstract authors (*p* = 0.544). This trend over time in female contribution was not identified in ASCO and ESMO presenters (*p* = 0.350 and *p* = 0.656).

## Discussion

Although gender differences have been acknowledged in medical research,^1^, ^2^, ^5^, ^6^, ^8^, ^9^ this is the first study to describe the gender gap in contribution to research presentations at the two largest oncological conferences in the world. Of all oncological studies presented at the main sessions of the past 8 ASCO Annual Meetings and 12 ESMO Congresses, the number of female presenters did not reach a quarter. In subsequently published papers, the share of female first and last authors was even smaller. The gender gap appears to be more prominent in oncological research than in clinical practice, because nearly half of the hematology–oncology fellowship trainees in the United States,^19^, ^20^ more than half of medical oncologists in several European countries^21^ and 37% of ASCO and 41% of ESMO members are female.^22^ Moreover, we found an association between sex of first author of subsequently published manuscripts and the journal's IF. Although IFs of these journals were all relatively high, which is not surprising given that these studies were presented at the most important sessions of the conferences, this corresponds with findings about the underrepresentation of female authors in high‐impact journals.^23^, ^24^

The lack of women presenting at oncological conferences is in line with the trend of gender differences in other research areas, where males numerically outweigh females, despite an increase in women entering scientific careers.^1^, ^2^, ^9^, ^25^, ^26^ The number of publications by male researchers remains significantly higher than those by females, as is also seen in authorships of oncological publications.^10^, ^12^ In our study, we found an overall female contribution to abstract authorships of 27–31%, with an increase of female contribution as abstract authors over time. However, this rise was not observed among female presenters at both conferences. Although it was not a statistically significant trend, the proportion of female presenters since 2015 appears to be shrinking rather than increasing and is therefore worrisome (Fig. 1).

Over the span of their academic career, publication productivity of women increases at a later stage of their career compared to men.^4^, ^27^ While the publication productivity of female researchers exceeded those of male researchers toward the end of their careers, that is, after 27 years of service, most leadership appointments occurred before the 20th year of service.^4^ Because productivity is an important factor in the selection of leaders, this could be one of the causes for the underrepresentation of women in leading positions. As not only the content of the abstract, but also past productivity and leadership positions may influence the selection of presenters for the most important sessions of ASCO and ESMO conferences, this could partly explain the underrepresentation of female presenters in these sessions as well.

Interpretation of data on gender disparities, including our data, may be hindered by a Simpson's paradox, as described earlier.^28^, ^29^ This paradox implies that an apparent association can actually be a result of a third dependent factor. For example, a finding that female researchers received requested grants less often than men was biased because women applied more often for grants in more competitive research fields.^28^ More specifically, our findings could be the result of self‐selection, in case that less women chose to submit an abstract to ASCO and ESMO or indicated they wanted to give a poster presentation rather than an oral presentation. In other scientific fields, gender differences in presentations at a congress have been identified as a result of self‐selection.^14^, ^17^, ^30^ For example, in biology women were asked less often as an invited speaker, even when adjusted for career stage, but also declined invitations more often than men.^17^ Similarly, at an anthropology conference, women appeared to ask for oral presentations less frequently than men, resulting in significantly more poster and less oral presentations than male reseachers.^30^ At an conference on evolutionary biology, women presented for relatively shorter duration compared to men despite a fifty‐fifty attendance, mainly because men requested longer presentations more often.^14^ Unfortunately, we did not have information about the number of submitted abstracts to ASCO and ESMO or whether the persons who submitted the abstracts requested a presentation or a poster. However, the findings in other fields highlight the possibility of self‐selection as a cause for the gender differences that we found and emphasize the need for women to increase their assertiveness in order to narrow the gender gap.

Gender, in contrast to sex, is a social construct of characteristics as norms and roles of and between women and men, instead of a “biological given” that is beyond our control.^31^, ^32^ To open up avenues for change, possible consequences of gender and its behavior‐based cause must be underlined.^33^ This starts with recognizing the gender gap^34^ and efforts to change perceptions of inequality associated with gender, for example, on competence^32^, ^35^ and meritocracy.^24^, ^27^, ^35^ Possible solutions beside acknowledgement of these biases that could bridge the gap in (oncological) research and level the playing field for both sexes may include encouragement of self‐promotion in female researchers, and implementation of guidelines that concern gender equality.^33^ For example, this could start with involving more women in the organizing committees of conferences, because this has been positively associated with female representation at conferences.^13^, ^30^ Second, the abstract assessment process could be changed by appraising the abstracts without information on the presenter's or authors' sexes or names. Moreover, female presenters could inspire and encourage female young researchers to follow their example. Finally, because all the female presenters came from the USA or Europe in our study, there should be greater awareness of the gender gap among researchers originating from other parts of the world.

Not only do gender gaps potentially disadvantage women, they could also impair patients outcomes and science.^1^ In oncological research, for example, several sex‐based differences in the treatment and outcomes of cancer patients have been explored and revealed important issues in, for example, drug responses and toxicity.^36^, ^37^, ^38^ The presence of a female author in a study has been positively associated with the likelihood of the exploration and analysis of these sex‐based differences.^39^, ^40^ Diversity in sex of researchers could therefore also contribute to a more diverse perception of science, possibly contributing to favorable outcomes for patients in the end, especially in the light of recent findings in sex‐based differences in oncology.^36^

Our study has some limitations. We only included abstracts presented at the most important sessions of two main oncological conferences in the world, therefore we do not know the gender balance in abstracts presented in other sessions or at other conferences. Moreover, a considerable part of the abstracts presented in 2018 were not yet published, which could have resulted in a bias. Lastly, we did not have data on the sex distribution of attendees at the conferences, or the proportion of females that participate in oncological research worldwide to compare this to the share of female presenters and abstract authors.

In conclusion, the share of female presenters at the main sessions of ASCO Annual Meetings and ESMO Congresses is only 21%, and 28% in authorships of these presented abstracts. Greater visibility of women at these large oncological conferences should be encouraged to allow acknowledgement for their research and opportunities for their academic career, as well as positively drive heterogeneity in research through diversity in sex of researches.

## Supporting information


**Appendix S1**: Supporting Information
