# Supplementary material for: From presentation to paper: Gender disparities in oncological research
Source: Int J Cancer. 2019 Oct 11;146(11):3011–21. doi: 10.1002/ijc.32660 (PMC7187424; doi:10.1002/ijc.32660)
Supplement: Supplementary file 1 — Appendix S1: Supporting Information [file IJC-146-3011-s001.docx]

**Supplementary Material – References ASCO and ESMO abstracts and papers**

41. Joensuu H, Eriksson M, Hatrmann J, Hall KS, Schutte J, Reichardt A, Schlemmer M, Wardelmann E, Ramadori G, Al-Batran S, Nilsson BE, Monge O, et al. Twelve versus 36 months of adjuvant imatinib (IM) as treatment of operable GIST with a high risk of recurrence: Final results of a randomized trial (SSGXVIII/AIO). *J Clin Oncol* 2011;29:1.

42. Joensuu H, Eriksson M, Hall KS, Hartmann T, Pink D, Schütte R, Ramadori G, Hohenberger P. One vs Three Years of Adjuvant Imatinib. *JAMA - J Am Med Assoc [Internet]* 2012;307:1265–72. Available from: http://dx.doi.org/10.1001/jama.2012.347

43. Ladenstein RL, Poetschger U, Luksch R, Brock P, Castel V, Yaniv I, Papadakis V, Laureys G, Malis J, Balwierz W, Ruud E, Kogner P, et al. Busulphan-melphalan as a myeloablative therapy (MAT) for high-risk neuroblastoma: Results from the HR-NBL1/SIOPEN trial. *J Clin Oncol [Internet]* 2011;29:2. Available from: https://doi.org/10.1200/jco.2011.29.18_suppl.2

44. Ladenstein R, Potschger U, Pearson ADJ, Brock P, Luksch R, Castel V, Yaniv I, Papadakis V, Laureys G, Malis J, Balwierz W, Ruud E, et al. Busulfan and melphalan versus carboplatin, etoposide, and melphalan as high-dose chemotherapy for high-risk neuroblastoma (HR-NBL1/SIOPEN): an international, randomised, multi-arm, open-label, phase 3 trial. *Lancet Oncol* 2017;18:500–14.

45. Larsen EC, Salzer WL, Devidas M, Nachman JB, Raetz EA, Loh ML, Heerema NA, Carroll AJ, Gastier-Foster JM, Borowitz MJ, Wood BL, Willman CL, et al. Comparison of high-dose methotrexate (HD-MTX) with Capizzi methotrexate plus asparaginase (C-MTX/ASNase) in children and young adults with high-risk acute lymphoblastic leukemia (HR-ALL): A report from the Children’s Oncology Group Study AALL0232. *J Clin Oncol [Internet]* 2011;29:3. Available from: http://ascopubs.org/doi/10.1200/jco.2011.29.18_suppl.3

46. Larsen EC, Devidas M, Chen S, Salzer WL, Raetz EA, Loh ML, Mattano LA, Cole C, Eicher A, Haugan M, Sorenson M, Heerema NA, et al. Dexamethasone and High-Dose Methotrexate Improve Outcome for Children and Young Adults With High-Risk B-Acute Lymphoblastic Leukemia: A Report From Children’s Oncology Group Study AALL0232. *J Clin Oncol [Internet]* 2016;34:2380–8. Available from: https://doi.org/10.1200/JCO.2015.62.4544

47. Chapman P, Hauschild A, Robert C, Larkin JMG, Haanen JBAG, Ribas A, Hogg D, O’Day S, Ascierto PA, Testori A, Lorigan P, Dummer R, et al. Phase III randomized, open-label, multicenter trial (BRIM3) comparing BRAF inhibitor vemurafenib with dacarbazine (DTIC) in patients with V600EBRAF-mutated melanoma. *J Clin Oncol* 2011;29:4.

48. Chapman PB, Hauschild A, Robert C, Haanen JB, Ascierto P, Larkin J, Dummer R, Garbe C, Testori A, Maio M, Hogg D, Lorigan P, et al. Improved Survival with Vemurafenib in Melanoma with BRAF V600E Mutation. *N Engl J Med [Internet]* 2011;364:2507–16. Available from: http://www.nejm.org/doi/abs/10.1056/NEJMoa1103782

49. Wolchok JD, Thomas L, Bondarenko IN, O’Day S, Weber JS, Garbe C, Francis S, Ibrahim RA, Hoos A, Robert C. Phase III randomized study of ipilimumab (IPI) plus dacarbazine (DTIC) versus DTIC alone as first-line treatment in patients with unresectable stage III or IV melanoma. *J Clin Oncol* 2011;29:5.

50. Robert C, Thomas L, Bondarenko I, O’Day S, Weber J, Garbe C, Lebbe C, Baurain J-F, Testori A, Grob J-J, Davidson N, Richards J, et al. Ipilimumab plus Dacarbazine for Previously Untreated Metastatic Melanoma. *N Engl J Med [Internet]* 2011;364:2517–26. Available from: http://www.nejm.org/doi/abs/10.1056/NEJMoa1104621

51. Blackwell, KM; Miles, D; Gianni, L; Krop, IE; Welslau, M; Baselga J et al. Primary results from EMILIA, a phase III study of trastuzumab emtansine (T-DMI) versus capecitabine (X) and lapatinib (L) in HER2-positive locally advanced with trastuzumab (T) and a taxane. *J Clin Oncol* 2012;30:1.

52. Verma S, Miles D, Gianni L, Krop IE, Welslau M, Baselga J, Pegram M, Oh D-Y, Dieras V, Guardino E, Fang L, Lu MW, et al. Trastuzumab Emtansine for HER2-Positive Advanced Breast Cancer. *N Engl J Med* 2012;367:1783–91.

53. Bent MJ Van Den, Hoang-xuan K, Brandes AA, Kros JM, Kouwenhoven MCM, Martin J, Taphoorn B, Delattre J, Bernsen HJJB, Frenay M, Tijssen C, Grisold W, et al. Long-term follow-up results of EORTC 26951 : A randomized phase III study on adjuvant PCV chemotherapy in anaplastic oligodendroglial tumors ( AOD ). Meeting : 2012 ASCO Annual Meeting Abstract No : 2000 First Author : Wolfgang Wick Category : Central Ner. *J Clin Oncol* 2012;30:2.

54. Van Den Bent MJ, Brandes AA, Taphoorn MJB, Kros JM, Kouwenhoven MCM, Delattre JY, Bernsen HJJA, Frenay M, Tijssen CC, Grisold W, Sipos L, Enting RH, et al. Adjuvant procarbazine, lomustine, and vincristine chemotherapy in newly diagnosed anaplastic oligodendroglioma: Long-term follow-up of EORTC brain tumor group study 26951. *J Clin Oncol* 2013;31:344–50.

55. Rummel MJ, Niederle N, Maschmeyer G, Banat AG, Von Gruenhagen U, Losem C, Dorothea KK, Heil G, Welslau M, Balser C, Kaiser U, Weidmann E, et al. Bendamustine plus rituximab (B-R) versus CHOP plus rituximab (CHOP-R) as first-line treatment in patients with indolent and mantle cell lymphomas (MCL): Updated results from the StiL NHL1 study. *J Clin Oncol [Internet]* 2012;30:3. Available from: http://www.embase.com/search/results?subaction=viewrecord&from=export&id=L71012630

56. Rummel MJ, Niederle N, Maschmeyer G, Banat GA, Von Grünhagen U, Losem C, Kofahl-Krause D, Heil G, Welslau M, Balser C, Kaiser U, Weidmann E, et al. Bendamustine plus rituximab versus CHOP plus rituximab as first-line treatment for patients with indolent and mantle-cell lymphomas: An open-label, multicentre, randomised, phase 3 non-inferiority trial. *Lancet* 2013;381:1203–10.

57. Hussain M, Tangen CM, Higano CS, Crawford ED, Liu G, Wilding G, Prescott S, Akdas A, Small EJ, Dawson NA, Donnelly BJ, Venner P, et al. Intermittent (IAD) versus continuous androgen deprivation (CAD) in hormone sensitive metastatic prostate cancer (HSM1PC) patients (pts): Results of S9346 (INT-0162), an international phase III trial. *J Clin Oncol* 2012;30:4.

58. Hussain M, Tangen CM, Berry DL, Higano CS, Crawford ED, Liu G, Wilding G, Prescott S, Sundaram SK, Small EJ, Dawson NA, Donnelly BJ, et al. Intermittent versus Continuous Androgen Deprivation in Prostate Cancer. *N Engl J Med* 2013;368:1314–25.

59. Gilbert MR, Dignam J, Won M, Blumenthal DT, Vogelbaum MA, Aldape KD, Colman H, Chakravarti A, Jeraj R, Armstrong TS, Wefel JS, Brown PD, et al. RTOG 0825: Phase III double-blind placebo-controlled trial evaluating bevacizumab (Bev) in patients (Pts) with newly diagnosed glioblastoma (GBM). *J Clin Oncol [Internet]* 2013;31:1. Available from: http://ascopubs.org/doi/10.1200/jco.2013.31.18_suppl.1

60. Gilbert MR, Dignam JJ, Armstrong TS, Wefel JS, Blumenthal DT, Vogelbaum MA, Colman H, Chakravarti A, Pugh S, Won M, Jeraj R, Brown PD, et al. A Randomized Trial of Bevacizumab for Newly Diagnosed Glioblastoma. *N Engl J Med [Internet]* 2014;370:699–708. Available from: http://www.nejm.org/doi/10.1056/NEJMoa1308573

61. Shastri SS, Mittra I, Mishra G, Gupta S, Dikshit R, Badwe RA. Effect of visual inspection with acetic acid (VIA) screening by primary health workers on cervical cancer mortality: A cluster randomized controlled trial in Mumbai India. *J Clin Oncol* 2013;31:2.

62. Shastri SS, Mittra I, Mishra GA, Gupta S, Dikshit R, Singh S, Badwe RA. Effect of VIA screening by primary health workers: Randomized controlled study in Mumbai, India. *J Natl Cancer Inst* 2014;106.

63. Tewari KS, Sill M, Long HJ, Ramondetta LM, Landrum LM, Oaknin A, Reid TJ, Leitao MM, Michael HE, Monk BJ. Incorporation of bevacizumab in the treatment of recurrent and metastatic cervical cancer: A phase III randomized trial of the Gynecologic Oncology Group. *J Clin Oncol* 2013;31:3.

64. Tewari KS, Sill MW, Long III HJ, Penson RT, Huang H, Ramondetta LM, Landrum LM, Oaknin A, Reid TJ, Leitao MM, Michael HE, Monk BJ. Improved Survival with Bevacizumab in Advanced Cervical Cancer. *N Engl J Med* 2014;370:734–43.

65. Brose MS, Nutting C, Jarzab B, Elisei R, Siena S, Bastholt L, de la Fouchardiere C, Pacini F, Paschke R, Shong YK, Sherman SI, Smit JWA, et al. Sorafenib in locally advanced or metastatic patients with radioactive iodine-refractory differentiated thyroid cancer: The phase III DECISION trial. *J Clin Oncol [Internet]* 2013;31:4. Available from: https://doi.org/10.1200/jco.2013.31.18_suppl.4

66. Brose MS, Nutting CM, Jarzab B, Elisei R, Siena S, Bastholt L, de la Fouchardiere C, Pacini F, Paschke R, Shong YK, Sherman SI, Smit JWA, et al. Sorafenib in radioactive iodine-refractory, locally advanced or metastatic differentiated thyroid cancer: a randomised, double-blind, phase 3 trial. *Lancet [Internet]* 2014;384:319–28. Available from: https://doi.org/10.1016/S0140-6736(14)60421-9

67. Gray RG, Rea D, Handley K, Bowden SJ, Perry P, Earl HM, Poole CJ, Bates T, Chetiyawardana S, Dewar JA, Fernando IN, Grieve R, et al. aTTom: Long-term effects of continuing adjuvant tamoxifen to 10 years versus stopping at 5 years in 6,953 women with early breast cancer. *J Clin Oncol [Internet]* 2013;31:5. Available from: https://doi.org/10.1200/jco.2013.31.18_suppl.5

68. Pagani O, Regan MM, Walley B, Fleming GF, Colleoni M, Lang I, Gomez HL, Tondini C, Burstein HJ, Perez EA, Ciruelos E, Stearns V, et al. Randomized comparison of adjuvant aromatase inhibitor (AI) exemestane (E) plus ovarian function suppression (OFS) vs tamoxifen (T) plus OFS in premenopausal women with hormone receptor-positive (HR+) early breast cancer (BC): Joint analysis of IBCSG TEXT. *J Clin Oncol [Internet]* 2014;32:LBA1–LBA1. Available from: https://doi.org/10.1200/jco.2014.32.15_suppl.lba1

69. Pagani O, Regan MM, Walley BA, Fleming GF, Colleoni M, Láng I, Gomez HL, Tondini C, Burstein HJ, Perez EA, Ciruelos E, Stearns V, et al. Adjuvant Exemestane with Ovarian Suppression in Premenopausal Breast Cancer. *N Engl J Med [Internet]* 2014;371:107–18. Available from: http://www.nejm.org/doi/10.1056/NEJMoa1404037

70. Sweeney C, Chen Y-H, Carducci MA, Liu G, Jarrard DF, Eisenberger MA, Wong Y-N, Hahn NM, Kohli M, Vogelzang NJ, Cooney MM, Dreicer R, et al. Impact on overall survival (OS) with chemohormonal therapy versus hormonal therapy for hormone-sensitive newly metastatic prostate cancer (mPrca): An ECOG-led phase III randomized trial. *J Clin Oncol* 2014;32:LBA2–LBA2.

71. Sweeney CJ, Chen Y-H, Carducci M, Liu G, Jarrard DF, Eisenberger M, Wong Y-N, Hahn N, Kohli M, Cooney MM, Dreicer R, Vogelzang NJ, et al. Chemohormonal Therapy in Metastatic Hormone-Sensitive Prostate Cancer. *N Engl J Med [Internet]* 2015;373:737–46. Available from: https://doi.org/10.1056/NEJMoa1503747

72. Venook AP, Niedzwiecki D, Lenz H-J, Innocenti F, Mahoney MR, O’Neil BH, Shaw JE, Polite BN, Hochster HS, Atkins JN, Goldberg RM, Mayer RJ, et al. CALGB/SWOG 80405: Phase III trial of irinotecan/5-FU/leucovorin (FOLFIRI) or oxaliplatin/5-FU/leucovorin (mFOLFOX6) with bevacizumab (BV) or cetuximab (CET) for patients (pts) with KRAS wild-type (wt) untreated metastatic adenocarcinoma of the colon or re. *J Clin Oncol [Internet]* 2014;32:LBA3–LBA3. Available from: https://doi.org/10.1200/jco.2014.32.15_suppl.lba3

73. Venook AP, Niedzwiecki D, Lenz HJ, Innocenti F, Fruth B, Meyerhardt JA, Schrag D, Greene C, O’Neil BH, Atkins JN, Berry S, Polite BN, et al. Effect of first-line chemotherapy combined with cetuximab or bevacizumab on overall survival in patients with KRAS wild-type advanced or metastatic colorectal cancer a randomized clinical trial. *JAMA - J Am Med Assoc* 2017;317:2392–401.

74. Piccart-Gebhart MJ, Holmes AP, Baselga J, De Azambuja E, Dueck AC, Viale G, Zujewski JA, Goldhirsch A, Santillana S, Pritchard KI, Wolff AC, Jackisch C, et al. First results from the phase III ALTTO trial (BIG 2-06; NCCTG [Alliance] N063D) comparing one year of anti-HER2 therapy with lapatinib alone (L), trastuzumab alone (T), their sequence (T→L), or their combination (T+L) in the adjuvant treatment of HER2-pos. *J Clin Oncol [Internet]* 2014;32:LBA4–LBA4. Available from: https://doi.org/10.1200/jco.2014.32.18_suppl.lba4

75. Wolchok JD, Chiarion-Sileni V, Gonzalez R, Rutkowski P, Grob JJ, Cowey CL, Lao CD, Schadendorf D, Ferrucci PF, Smylie M, Dummer R, Hill AG, et al. Efficacy and safety results from a phase III trial of nivolumab (NIVO) alone or combined with ipilimumab (IPI) versus IPI alone in treatment-naive patients (pts) with advanced melanoma (MEL) (CheckMate 067). *J Clin Oncol [Internet]* 2015;33:LBA1–LBA1. Available from: https://doi.org/10.1200/jco.2015.33.18_suppl.lba1

76. Larkin J, Chiarion-Sileni V, Gonzalez R, Grob JJ, Cowey CL, Lao CD, Schadendorf D, Dummer R, Smylie M, Rutkowski P, Ferrucci PF, Hill A, et al. Combined Nivolumab and Ipilimumab or Monotherapy in Untreated Melanoma. *N Engl J Med [Internet]* 2015;373:23–34. Available from: https://doi.org/10.1056/NEJMoa1504030

77. Armstrong GT, Yasui Y, Chen Y, Leisenring WM, Gibson TM, Mertens A, Stovall M, Hudson MM, Oeffinger KC, Bhatia S, Krull KR, Nathan PC, et al. Reduction in late mortality among 5-year survivors of childhood cancer: A report from the Childhood Cancer Survivor Study (CCSS). *J Clin Oncol* 2015;33:LBA2–LBA2.

78. Armstrong GT, Chen Y, Yasui Y, Leisenring W, Gibson TM, Mertens AC, Stovall M, Oeffinger KC, Bhatia S, Krull KR, Nathan PC, Neglia JP, et al. Reduction in Late Mortality among 5-Year Survivors of Childhood Cancer. *N Engl J Med [Internet]* 2016;374:833–42. Available from: https://doi.org/10.1056/NEJMoa1510795

79. D’Cruz A, Dandekar M, Vaish R, Arya S, Pantvaidya G, Chaturvedi P, Chaukar D, Pai PS, Deshmukh A, Kane S, Nair D, Nair SV, et al. Elective versus therapeutic neck dissection in the clinically node negative early oral cancer: A randomised control trial (RCT). *J Clin Oncol [Internet]* 2015;33:LBA3–LBA3. Available from: https://doi.org/10.1200/jco.2015.33.18_suppl.lba3

80. D’Cruz AK, Vaish R, Kapre N, Dandekar M, Gupta S, Hawaldar R, Agarwal JP, Pantvaidya G, Chaukar D, Deshmukh A, Kane S, Arya S, et al. Elective versus Therapeutic Neck Dissection in Node-Negative Oral Cancer. *N Engl J Med [Internet]* 2015;373:521–9. Available from: https://doi.org/10.1056/NEJMoa1506007

81. Brown PD, Asher AL, Ballman K V., Farace E, Cerhan JH, Anderson SK, Carrero XW, Barker FG, Deming RL, Burri S, Menard C, Chung C, et al. NCCTG N0574 (Alliance): A phase III randomized trial of whole brain radiation therapy (WBRT) in addition to radiosurgery (SRS) in patients with 1 to 3 brain metastases. *J Clin Oncol [Internet]* 2015;33:LBA4–LBA4. Available from: http://ascopubs.org/doi/10.1200/jco.2015.33.18_suppl.lba4

82. Brown PD, Jaeckle K, Ballman K V., Farace E, Cerhan JH, Keith Anderson S, Carrero XW, Barker FG, Deming R, Burri SH, Ménard C, Chung C, et al. Effect of radiosurgery alone vs radiosurgery with whole brain radiation therapy on cognitive function in patients with 1 to 3 brain metastases a randomized clinical trial. *JAMA - J Am Med Assoc [Internet]* 2016;316:401–9. Available from: +

83. Goss PE, Ingle JN, Pritchard KI, Robert NJ, Muss H, Gralow J, Gelmon KA, Whelan TJ, Strasser-Weippl K, Rubin S, Sturtz K, Wolff AC, et al. A randomized trial (MA.17R) of extending adjuvant letrozole for 5 years after completing an initial 5 years of aromatase inhibitor therapy alone or preceded by tamoxifen in postmenopausal women with early-stage breast cancer. *J Clin Oncol [Internet]* 2016;34:LBA1–LBA1. Available from: http://ascopubs.org/doi/10.1200/JCO.2016.34.18_suppl.LBA1

84. Goss PE, Ingle JN, Pritchard KI, Robert NJ, Muss H, Gralow J, Gelmon K, Whelan T, Strasser-Weippl K, Rubin S, Sturtz K, Wolff AC, et al. Extending Aromatase-Inhibitor Adjuvant Therapy to 10 Years. *N Engl J Med [Internet]* 2016;375:209–19. Available from: http://www.nejm.org/doi/10.1056/NEJMoa1604700

85. Perry JR, Laperriere N, O’Callaghan CJ, Brandes AA, Menten J, Phillips C, Fay MF, Nishikawa R, Cairncross JG, Roa W, Osoba D, Sahgal A, et al. A phase III randomized controlled trial of short-course radiotherapy with or without concomitant and adjuvant temozolomide in elderly patients with glioblastoma (CCTG CE.6, EORTC 26062-22061, TROG 08.02, NCT00482677). *J Clin Oncol [Internet]* 2016;34:LBA2–LBA2. Available from: http://ascopubs.org/doi/10.1200/JCO.2016.34.18_suppl.LBA2

86. Perry JR, Laperriere N, O’Callaghan CJ, Brandes AA, Menten J, Phillips C, Fay M, Nishikawa R, Cairncross JG, Roa W, Osoba D, Rossiter JP, et al. Short-Course Radiation plus Temozolomide in Elderly Patients with Glioblastoma. *N Engl J Med [Internet]* 2017;376:1027–37. Available from: http://www.nejm.org/doi/10.1056/NEJMoa1611977

87. Park JR, Kreissman SG, London WB, Naranjo A, Cohn SL, Hogarty MD, Tenney SC, Haas-Kogan D, Shaw PJ, Geiger JD, Doski JJ, Gorges SW, et al. A phase III randomized clinical trial (RCT) of tandem myeloablative autologous stem cell transplant (ASCT) using peripheral blood stem cell (PBSC) as consolidation therapy for high-risk neuroblastoma (HR-NB): A Children’s Oncology Group (COG) study. *J Clin Oncol [Internet]* 2016;34:LBA3–LBA3. Available from: http://ascopubs.org/doi/10.1200/JCO.2016.34.18_suppl.LBA3

88. Palumbo A, Chanan-Khan AAA, Weisel K, Nooka AK, Masszi T, Beksac M. Phase III randomized controlled study of daratumumab, bortezomib, and dexamethasone (DVd) versus bortezomib and dexamethasone (Vd) in patients (pts) with relapsed or refractory multiple myeloma (RRMM): CASTOR study. *J Clin Oncol* 2016;34:LBA4–LBA4.

89. Palumbo A, Chanan-Khan A, Weisel K, Nooka AK, Masszi T, Beksac M, Spicka I, Hungria V, Munder M, Mateos M V, Mark TM, Qi M, et al. Daratumumab, Bortezomib, and Dexamethasone for Multiple Myeloma. *N Engl J Med* 2016;375:754–66.

90. Shi Q, Sobrero AF, Shields AF, Yoshino T, Paul J, Taieb J, Sougklakos I, Kerr R, Labianca R, Meyerhardt JA, Bonnetain F, Watanabe T, et al. Prospective pooled analysis of six phase III trials investigating duration of adjuvant (adjuv) oxaliplatin-based therapy (3 vs 6 months) for patients (pts) with stage III colon cancer (CC): The IDEA (International Duration Evaluation of Adjuvant chemother. *J Clin Oncol [Internet]* 2017;35:LBA1–LBA1. Available from: https://doi.org/10.1200/JCO.2017.35.18_suppl.LBA1

91. Grothey A, Sobrero AF, Shields AF, Yoshino T, Paul J, Taieb J, Souglakos J, Shi Q, Kerr R, Labianca R, Meyerhardt JA, Vernerey D, et al. Duration of Adjuvant Chemotherapy for Stage III Colon Cancer. *N Engl J Med [Internet]* 2018;378:1177–88. Available from: https://doi.org/10.1056/NEJMoa1713709

92. Basch EM, Deal AM, Dueck AC, Bennett AV, Atkinson TM, Scher HI, Kris MG, Hudis CA, Sabbatini P, Dulko D, Rogak LJ, Barz AE, et al. Overall survival results of a randomized trial assessing patient-reported outcomes for symptom monitoring during routine cancer treatment. *J Clin Oncol [Internet]* 2017;35:LBA2–LBA2. Available from: https://doi.org/10.1200/JCO.2017.35.18_suppl.LBA2

93. Basch E, Deal AM, Dueck AC, Scher HI, Kris MG, Hudis C, Schrag D. Overall survival results of a trial assessing patient-reported outcomes for symptom monitoring during routine cancer treatment. *JAMA - J Am Med Assoc* 2017;318:197–8.

94. Fizazi K, Tran N, Fein LE, Matsubara N, Antolin AR, Alekseev BY, Ozguroglu M, Ye D, Feyerabend S, Protheroe A, De Porre P, Kheoh T, et al. LATITUDE: A phase III, double-blind, randomized trial of androgen deprivation therapy with abiraterone acetate plus prednisone or placebos in newly diagnosed high-risk metastatic hormone-naive prostate cancer. *J Clin Oncol* 2017;35:LBA3–LBA3.

95. Fizazi K, Tran N, Fein L, Matsubara N, Rodriguez-Antolin A, Alekseev BY, Özgüroğlu M, Ye D, Feyerabend S, Protheroe A, De Porre P, Kheoh T, et al. Abiraterone plus Prednisone in Metastatic, Castration-Sensitive Prostate Cancer. *N Engl J Med [Internet]* 2017;377:352–60. Available from: https://doi.org/10.1056/NEJMoa1704174

96. Robson ME, Im S-A, Senkus E, Xu B, Domchek SM, Masuda N, Delaloge S, Li W, Tung NM, Armstrong A, Wu W, Goessl CD, et al. OlympiAD: Phase III trial of olaparib monotherapy versus chemotherapy for patients (pts) with HER2-negative metastatic breast cancer (mBC) and a germline BRCA mutation (gBRCAm). *J Clin Oncol* 2017;35:LBA4–LBA4.

97. Robson M, Im S-A, Senkus E, Xu B, Domchek SM, Masuda N, Delaloge S, Li W, Tung N, Armstrong A, Wu W, Goessl C, et al. Olaparib for Metastatic Breast Cancer in Patients with a Germline *BRCA* Mutation. *N Engl J Med [Internet]* 2017;377:523–33. Available from: http://www.nejm.org/doi/10.1056/NEJMoa1706450

98. Sparano JA, Gray RJ, Wood WC, Makower DF, Lively TG, Saphner TJ, Keane MM, Gomez HL, Reddy PS, Goggins TF, Mayer IA, Toppmeyer D, et al. TAILORx: Phase III trial of chemoendocrine therapy versus endocrine therapy alone in hormone receptor-positive, HER2-negative, node-negative breast cancer and an intermediate prognosis 21-gene recurrence score. *J Clin Oncol [Internet]* 2018;36:LBA1–LBA1. Available from: https://doi.org/10.1200/JCO.2018.36.18_suppl.LBA1

99. Sparano JA, Gray RJ, Makower DF, Pritchard KI, Albain KS, Hayes DF, Geyer CE, Dees EC, Goetz MP, Olson JA, Lively T, Badve SS, et al. Adjuvant Chemotherapy Guided by a 21-Gene Expression Assay in Breast Cancer. *N Engl J Med [Internet]* 2018;379:111–21. Available from: https://doi.org/10.1056/NEJMoa1804710

100. Bisogno G, De Salvo GL, Bergeron C, Jenney M, Merks JHM, Minard-Colin V, Orbach D, Glosli H, Chisholm J, Casanova M, Gallego Melcon S, Ferrari A. Maintenance low-dose chemotherapy in patients with high-risk (HR) rhabdomyosarcoma (RMS): A report from the European Paediatric Soft Tissue Sarcoma Study Group (EpSSG). *J Clin Oncol [Internet]* 2018;36:LBA2–LBA2. Available from: https://doi.org/10.1200/JCO.2018.36.18_suppl.LBA2

101. Bisogno G, Jenney M, Bergeron C, Gallego Melcon S, Ferrari A, Oberlin O, Carli M, Stevens M, Kelsey A, De Paoli A, Gaze MN, Martelli H, et al. Addition of dose-intensified doxorubicin to standard chemotherapy for rhabdomyosarcoma (EpSSG RMS 2005): a multicentre, open-label, randomised controlled, phase 3 trial. *Lancet Oncol* 2018;19:1061–71.

102. Mejean A, Escudier B, Thezenas S, Beauval J-B, Geoffroy L, Bensalah K, Thiery-Vuillemin A, Cormier L, Lang H, Guy L, Gravis G, Rolland F, et al. CARMENA: Cytoreductive nephrectomy followed by sunitinib versus sunitinib alone in metastatic renal cell carcinoma—Results of a phase III noninferiority trial. *J Clin Oncol [Internet]* 2018;36:LBA3–LBA3. Available from: https://doi.org/10.1200/JCO.2018.36.18_suppl.LBA3

103. Méjean A, Ravaud A, Thezenas S, Colas S, Beauval J-B, Bensalah K, Geoffrois L, Thiery-Vuillemin A, Cormier L, Lang H, Guy L, Gravis G, et al. Sunitinib Alone or after Nephrectomy in Metastatic Renal-Cell Carcinoma. *N Engl J Med [Internet]* 2018;379:417–27. Available from: https://doi.org/10.1056/NEJMoa1803675

104. Lopes G, Wu Y-L, Kudaba I, Kowalski D, Cho BC, Castro G, Srimuninnimit V, Bondarenko I, Kubota K, Lubiniecki GM, Zhang J, Kush DA, et al. Pembrolizumab (pembro) versus platinum-based chemotherapy (chemo) as first-line therapy for advanced/metastatic NSCLC with a PD-L1 tumor proportion score (TPS) ≥ 1%: Open-label, phase 3 KEYNOTE-042 study. *J Clin Oncol [Internet]* 2018;36:LBA4–LBA4. Available from: https://doi.org/10.1200/JCO.2018.36.18_suppl.LBA4

105. Mok TSK, Wu YL, Kudaba I, Kowalski DM, Cho BC, Turna HZ, Castro G, Srimuninnimit V, Laktionov KK, Bondarenko I, Kubota K, Lubiniecki GM, et al. Pembrolizumab versus chemotherapy for previously untreated, PD-L1-expressing, locally advanced or metastatic non-small-cell lung cancer (KEYNOTE-042): a randomised, open-label, controlled, phase 3 trial. *Lancet* 2019;393:1819–30.

106. Manegold C, von Pawel J, Zatloukal P, Ramlau R, Gorbounova V, Hirsh V, Leighl N, Mezger J, Archer V, Reck M. BO177701 (AVAIL): A phase III randomised study of first-line bevacizumab combined with cisplatin/gemcitabine (CG) in patients (PTS) with advanced or recurrent non-squamous, non-small cell lung cancer (NSCLC). *Ann Oncol* 2008;19:1.

107. Reck M, Von Pawel J, Zatloukal P, Ramlau R, Gorbounova V, Hirsh V, Leighl N, Mezger J, Archer V, Moore N, Manegold C. Phase III trial of cisplatin plus gemcitabine with either placebo or bevacizumab as first-line therapy for nonsquamous non-small-cell lung cancer: AVAiL. *J Clin Oncol* 2009;27:1227–34.

108. Mok T, Wu Y-L, Thongprasert S, Yang C-H, Chu D, Saijo N, Jiang H, Watkins C, Armour A, Fukuoka M. PHASE III, RANDOMISED, OPEN-LABEL, FIRST-LINE STUDY OF GEFITINIB (G) VS CARBOPLATIN/PACLITAXEL (C/P) IN CLINICALLY SELECTED PATIENTS (PTS) WITH ADVANCED NON-SMALL-CELL LUNG CANCER (NSCLC) (IPASS). *Ann Oncol* 2008;19:1.

109. Mok TS, Wu Y-L, Thongprasert S, Yang C-H, Chu D-T, Saijo N, Sunpaweravong P, Han B, Margono B, Ichinose Y, Nishiwaki Y, Ohe Y, et al. Gefitinib or Carboplatin–Paclitaxel in Pulmonary Adenocarcinoma. *N Engl J Med [Internet]* 2009;361:947–57. Available from: http://www.nejm.org/doi/abs/10.1056/NEJMoa0810699

110. Midgley RSJ, McConkey CC, Langman MJ, Smith JL, Julier P, Iveson C, Johnstone E, Chen X, Dunn JA, Kerr DJ, Grp VT. VICTOR: A PHASE III PLACEBO-CONTROLLED TRIAL OF ROFECOXIB IN COLORECTAL CANCER PATIENTS FOLLOWING SURGICAL RESECTION. *Ann Oncol* 2008;19:1.

111. Midgley RS, McConkey CC, Johnstone EC, Dunn JA, Smith JL, Grumett SA, Julier P, Iveson C, Yanagisawa Y, Warren B, Langman MJ, Kerr DJ. Phase III randomized trial assessing rofecoxib in the adjuvant setting of colorectal cancer: Final results of the VICTOR trial. *J Clin Oncol* 2010;28:4575–80.

112. Monk BJ, Herzog T, Kaye S, Krasner CN, Vermorken J, Muggia F, Pujade-Lourraine E, Renshaw FG, Lebedinsky C, Poveda A. A RANDOMIZED PHASE III STUDY OF TRABECTEDIN WITH PEGYLATED LIPOSOMAL DOXORUBICIN (PLD) VERSUS PLD IN RELAPSED, RECURRENT OVARIAN CANCER (OC). *Ann Oncol* 2008;19:2.

113. Monk BJ, Herzog TJ, Kaye SB, Krasner CN, Vermorken JB, Muggia FM, Pujade-Lauraine E, Lisyanskaya AS, Makhson AN, Rolski J, Gorbounova VA, Ghatage P, et al. Trabectedin plus pegylated liposomal doxorubicin in recurrent ovarian cancer. *J Clin Oncol* 2010;28:3107–14.

114. Lee KS, Ro J, Lee ES, Kang HS, Kim SW, Kwon Y, Shin KH, Kim EA. PHASE II STUDY OF PRIMARY SYSTEMIC THERAPY WITH WEEKLY PACLITAXEL PLUS GEMCITABINE IN PATIENTS WITH STAGE II AND III BREAST CANCER: FINAL ANALYSIS. *Ann Oncol* 2008;19:81.

115. Brada M, Stenning S, Gabe R, Thompson LC, Levy D, Rampling R, Erridge S, Saran F, Gattamaneni R, Hopkins K, Beall S, Collins VP, et al. Temozolomide Versus Procarbazine, Lomustine, and Vincristine in Recurrent High-Grade Glioma. *J Clin Oncol* 2010;28:4601–8.

116. Karapetis C, Khambata-Ford S, Jonker D, O’Callaghan C, Tu D, Vachan B, Simes J, Langer C, Moore M, Zalcberg J. KRAS MUTATION STATUS IS A PREDICTIVE BIOMARKER FOR CETUXIMAB BENEFIT IN THE TREATMENT OF ADVANCED COLORECTAL CANCER - RESULTS FROM NCIC CTG CO.17: A PHASE III TRIAL OF CETUXIMAB VERSUS BEST SUPPORTIVE CARE. *Ann Oncol* 2008;19:2.

117. Karapetis CS, Khambata-Ford S, Jonker DJ, O’Callaghan CJ, Tu D, Tebbutt NC, Simes RJ, Chalchal H, Shapiro JD, Robitaille S, Price TJ, Shepherd L, et al. K-ras mutations and benefit from cetuximab in advanced colorectal cancer. *N Engl J Med* 2008;359:1757–65.

118. Loehr M, Haas S, Bechstein W-O, Bodoky G, Jaeger D, Karrasch M, Mescheder A, Meyer I, Pap A, Foelsch U. 12-MONTH SURVIVAL DATA OF A PHASE II TRIAL: FIRST LINE TREATMENT OF INOPERABLE PANCREATIC ADENOCARCINOMA WITH CATIONIC LIPID COMPLEXED PACLITAXEL NANOPARTICLES (ENDOTAG-1 (R)) PLUS GEMCITABINE COMPARED WITH GEMCITABINE MONOTHERAPY. *Ann Oncol* 2008;19:2–3.

119. Löhr JM, Haas SL, Bechstein WO, Bodoky G, Cwiertka K, Fischbach W, Fölsch UR, Jäger D, Osinsky D, Prausova J, Schmidt WE, Lutz MP, et al. Cationic liposomal paclitaxel plus gemcitabine or gemcitabine alone in patients with advanced pancreatic cancer: A randomized controlled phase II trial. *Ann Oncol* 2012;23:1214–22.

120. Patel PM, Suciu S, Mortier L, Kruit W, Robert C, Schadendorf D, Keilholz U, Musat E, Eggermont A, Spatz A. EXTENDED SCHEDULE, ESCALATED DOSE TEMOZLOMIDE VERSUS DACARBAZINE IN STAGE IV MALIGNANT MELANOMA: FINAL RESULTS OF THE RANDOMISED PHASE III STUDY(EORTC 18032). *Ann Oncol* 2008;19:3.

121. Patel PM, Suciu S, Mortier L, Kruit WH, Robert C, Schadendorf D, Trefzer U, Punt CJA, Dummer R, Davidson N, Becker J, Conry R, et al. Extended schedule, escalated dose temozolomide versus dacarbazine in stage IV melanoma: Final results of a randomised phase III study (EORTC 18032). *Eur J Cancer* 2011;47:1476–83.

122. Auerbach M, Silberstein PT, Webb RT, Averyanova S, Ciuleanu T-E, Cam L, Shao J, Lillie T. DARBEPOETIN ALFA (DA) 500MCG OR 300MCG ONCE EVERY THREE WEEKS (Q3W) WITH OR WITHOUT IRON IN PATIENTS (PTS) WITH CHEMOTHERAPY-INDUCED ANEMIA (CIA). *Ann Oncol* 2008;19:3.

123. Auerbach M, Silberstein PT, Webb RT, Averyanova S, Ciuleanu TE, Shao J, Bridges K. Darbepoetin alfa 300 or 500 μg once every 3 weeks with or without intravenous iron in patients with chemotherapy-induced anemia. *Am J Hematol* 2010;85:655–63.

124. Van Hemelrijck M, Garmo H, Bratt O, Bill-Axelson A, Lambe M, Stattin P, Holmberg L, Adolfsson J. Increased cardiovascular morbidity and mortality following endocrine treatment for prostate cancer: an analysis in 30,642 men in PCBaSe Sweden. *EJC Suppl* 2009;7:1.

125. Van Hemelrijck M, Garmo H, Holmberg L, Ingelsson E, Bratt O, Bill-Axelson A, Lambe M, Stattin P, Adolfsson J. Absolute and relative risk of cardiovascular disease in men with prostate cancer: Results from the population-based PCBaSe Sweden. *J Clin Oncol* 2010;28:3448–56.

126. van de Velde C, Seynaeve C, Hasenburg A, Rea D, Vannetzel JM, Paridaens R, Markopoulos C, Smeets J, Nortier JWR, Jones SE. Results of the TEAM (tamoxifen exemestane adjuvant multinational) prospective randomized phase III trial in hormone sensitive postmenopausal early breast cancer. *EJC Suppl* 2009;7:1.

127. Van De Velde CJ, Rea D, Seynaeve C, Putter H, Hasenburg A, Vannetzel JM, Paridaens R, Markopoulos C, Hozumi Y, Hille ET, Kieback DG, Asmar L, et al. Adjuvant tamoxifen and exemestane in early breast cancer (TEAM): A randomised phase 3 trial. *Lancet* 2011;377:321–31.

128. Brunt AM, Sydenham M, Bliss J, Coles C, Gothard L, Harnett A, Haviland J, Syndikus I, Wheatley D, Yarnold J. A 5-fraction regimen of adjuvant radiotherapy for women with early breast cancer: first analysis of the randomised UK FAST trial (ISRCTN62488883, CRUKE/04/015). *EJC Suppl* 2009;7:2.

129. Yarnold JR, Agrawal RK, Alhasso A, Barrett-Lee PJ, Bliss JM, Bliss P, Bloomfield D, Bowen J, Brunt AM, Donovan E, Emson M, Goodman A, et al. First results of the randomised UK FAST Trial of radiotherapy hypofractionation for treatment of early breast cancer (CRUKE/04/015). *Radiother Oncol* 2011;100:93–100.

130. Issels R, Lindner LH, Wendtner CM, Hohenberger P, Reichardt P, Daugaard S, Mansmann U, Hiddemann W, Blay JY, Verweij J. Impact of regional hyperthermia (RHT) on response to neo-adjuvant chemotherapy and survival of patients with high-risk soft-tissue sarcoma (HR-STS): Results of the randomized EORTC-ESHO intergroup trial (NCI-00003052). *EJC Suppl* 2009;7:2.

131. Issels RD, Lindner LH, Verweij J, Wust P, Reichardt P, Schem BC, Abdel-Rahman S, Daugaard S, Salat C, Wendtner CM, Vujaskovic Z, Wessalowski R, et al. Neo-adjuvant chemotherapy alone or with regional hyperthermia for localised high-risk soft-tissue sarcoma: A randomised phase 3 multicentre study. *Lancet Oncol* 2010;11:561–70.

132. Stopeck A, Body JJ, Fujiwara Y, Lipton A, Steger GG, Viniegra M, Fan M, Braun A, Dansey R, Jun S. Denosumab versus zoledronic acid for the treatment of breast cancer patients with bone metastases: results of a randomized phase 3 study. *EJC Suppl* 2009;7:2–3.

133. Stopeck AT, Lipton A, Body JJ, Steger GG, Tonkin K, De Boer RH, Lichinitser M, Fujiwara Y, Yardley DA, Viniegra M, Fan M, Jiang Q, et al. Denosumab compared with zoledronic acid for the treatment of bone metastases in patients with advanced breast cancer: A randomized, double-blind study. *J Clin Oncol* 2010;28:5132–9.

134. van der Burg MEL, Rustin GJ. Randomized MRC OV05/EORTC 55955 trial in recurrent ovarian cancer: early treatment based on increased serum CA125 alone versus delayed treatment based on conventional clinical indicators. *EJC Suppl* 2009;7:3.

135. Rustin GJS, Van Der Burg MEL, Griffin CL, Guthrie D, Lamont A, Jayson GC, Kristensen G, Mediola C, Coens C, Qian W, Parmar MKB, Swart AM. Early versus delayed treatment of relapsed ovarian cancer (MRC OV05/EORTC 55955): A randomised trial. *Lancet* 2010;376:1155–63.

136. Steger GG, Greil R, Jakesz R, Lang A, Mlineritsch B, Melbinger-Zeinitzer E, Martin C, Samonigg H, Kubista E, Gnant M. A randomized phase III study comparing epirubicin, docetaxel, and capecitabine (EDC) to epirubicin and docetaxel (ED) as neoadjuvant treatment for early breast cancer - first results of the Austrian Breast and Colorectal Cancer Study Group-Trial 24 (ABCSG. *EJC Suppl* 2009;7:3.

137. Steger GG, Greil R, Lang A, Rudas M, Fitzal F, Mlineritsch B, Hartmann BL, Bartsch R, Melbinger E, Hubalek M, Stoeger H, Dubsky P, et al. Epirubicin and docetaxel with or without capecitabine as neoadjuvant treatment for early breast cancer: final results of a randomized phase III study (ABCSG-24). *Ann Oncol* 2014;25:366–71.

138. Baselga J, Segalla JGM, Roche H, del Giglio A, Ciruelos EM, Cabral Filho S, Gomez P, Lluch A, Llombart A, Costa F. SOLTI-0701: A double-blind, randomized phase 2b study evaluating the efficacy and safety of sorafenib (SOR) compared to placebo (PL) when administered in combination with capecitabine (CAP) in patients (pts) with locally advanced (adv) or metastatic (met). *EJC Suppl* 2009;7:3–4.

139. Baselga J, Segalla JGM, Roché H, Del Giglio A, Pinczowski H, Ciruelos EM, Cabral Filho S, Gómez P, Van Eyll B, Bermejo B, Llombart A, Garicochea B, et al. Sorafenib in combination with capecitabine: An oral regimen for patients with HER2-negative locally advanced or metastatic breast cancer. *J Clin Oncol* 2012;30:1484–91.

140. Baumann M, Herrmann T, Koch R, Wahlers B, Kepka L, Marschke G, Feltl D, Fietkau R, Budach V, Dunst J, Grp C. Final results of the randomized phase III CHARTWEL-trial (ARO 97-1) comparing hyperfractionated-accelerated vs conventionally fractionated radiotherapy in non-small cell lung cancer (NSCLC). *EJC Suppl* 2009;7:4.

141. Baumann M, Herrmann T, Koch R, Matthiessen W, Appold S, Wahlers B, Kepka L, Marschke G, Feltl D, Fietkau R, Budach V, Dunst J, et al. Final results of the randomized phase III CHARTWEL-trial (ARO 97-1) comparing hyperfractionated-accelerated versus conventionally fractionated radiotherapy in non-small cell lung cancer (NSCLC). *Radiother Oncol* 2011;100:76–85.

142. Hailer D, Tabernero J, Maroun J, de Braud F, Price T, Van Cutsem E, Hill M, Gilberg F, Rittweger K, Schmoll H. First efficacy findings from a randomized phase III trial of capecitabine plus oxaliplatin vs. bolus 5-FU/LV for stage III colon cancer (NO16968/XELOXA study). *EJC Suppl* 2009;7:4.

143. Schmoll HJ, Tabernero J, Maroun J, De Braud F, Price T, Van Cutsem E, Hill M, Hoersch S, Rittweger K, Haller DG. Capecitabine plus oxaliplatin compared with fluorouracil/folinic acid as adjuvant therapy for stage III colon cancer: Final Results of the NO16968 randomized controlled phase III trial. *J Clin Oncol* 2015;33:3733–40.

144. Maughan T, Adams RA, Smith CG, Seymour MT, Wilson R, Meade AM, Fisher D, Madi A, Cheadle J, Kaplan R. Addition of cetuximab to oxaliplatin-based combination chemotherapy (CT) in patients with KRAS wild-type advanced colorectal cancer (ACRC): a randomised superiority trial (MRC COIN). *EJC Suppl* 2009;7:4–5.

145. Maughan TS, Adams RA, Smith CG, Meade AM, Seymour MT, Wilson RH, Idziaszczyk S, Harris R, Fisher D, Kenny SL, Kay E, Mitchell JK, et al. Addition of cetuximab to oxaliplatin-based first-line combination chemotherapy for treatment of advanced colorectal cancer: Results of the randomised phase 3 MRC COIN trial. *Lancet* 2011;377:2103–14.

146. Badve S, Shen C, Thorat M, Li L, Gagnon R, Koehler M, Ellis C, O’Shaughnessy J, Baselga J, Sledge G. Identification of gene expression profiles that predict response to HER2-targeted therapy. *Eur J Cancer, Suppl [Internet]* 2009;7:5. Available from: http://www.embase.com/search/results?subaction=viewrecord&from=export&id=L70211449

147. Chapman P, Puzanov I, Sosman J, Kim K, Ribas A, McArthur G, Lee R, Grippo J, Nolop K, Flaherty K. Early efficacy signal demonstrated in advanced melanoma in a phase I trial of the oncogenic BRAF-selective inhibitor PLX4032. *EJC Suppl* 2009;7:5.

148. Flaherty KT, Puzanov I, Kim KB, Ribas A, McArthur GA, Sosman JA, O’Dwyer PJ, Lee RJ, Grippo JF, Nolop K, Chapman PB. Inhibition of Mutated, Activated BRAF in Metastatic Melanoma. *N Engl J Med [Internet]* 2010;363:809–19. Available from: http://www.nejm.org/doi/abs/10.1056/NEJMoa1002011

149. Johnson B, Miller V, Amler L, Stern H, Soh C, O’Connor P, Kabbinavar F. Biomarker evaluation in the randomized, double-blind, placebo-controlled, Phase IIIb ATLAS Trial, comparing bevacizumab (B) therapy with or without erlotinib (E), after completion of chemotherapy with B for the treatment of locally-advanced, recurrent, or. *EJC Suppl* 2009;7:5–6.

150. Johnson BE, Kabbinavar F, Fehrenbacher L, Hainsworth J, Kasubhai S, Kressel B, Lin CY, Marsland T, Patel T, Polikoff J, Rubin M, White L, et al. ATLAS: Randomized, double-blind, placebo-controlled, phase IIIB trial comparing bevacizumab therapy with or without erlotinib, after completion of chemotherapy, with bevacizumab for first-line treatment of advanced non-small-cell lung cancer. *J Clin Oncol* 2013;31:3926–34.

151. Inoue A, Kobayashi K, Maemondo M, Sugawara S, Oizumi S, Saijo Y, Genma A, Morita S, Hagiwara K, Nukiwa T. A randomized phase III study comparing gefitinib with carboplatin (CBDCA) plus paclitaxel (TXL) for the first-line treatment of non-small cell lung cancer (NSCLC) with sensitive EGFR mutations: NEJ002 study. *EJC Suppl* 2009;7:6.

152. Inoue A, Kobayashi K, Maemondo M, Sugawara S, Oizumi S, Isobe H, Gemma A, Harada M, Yoshizawa H, Kinoshita I, Fujita Y, Okinaga S, et al. Updated overall survival results from a randomized phase III trial comparing gefitinib with carboplatin-paclitaxel for chemo-naïve non-small cell lung cancer with sensitive EGFR gene mutations (NEJ002). *Ann Oncol* 2013;24:54–9.

153. Douillard J, Siena S, Cassidy J, Tabernero J, Burkes R, Barugel ME, Humblet Y, Cunningham D, Wolf M, Gansert JL. Randomized phase 3 study of panitumumab with FOLFOX4 compared to FOLFOX4 alone as 1(st)-line treatment (tx) for metastatic colorectal cancer (mCRC): the PRIME trial. *EJC Suppl* 2009;7:6.

154. Douillard JY, Siena S, Cassidy J, Tabernero J, Burkes R, Barugel M, Humblet Y, Bodoky G, Cunningham D, Jassem J, Rivera F, Kocákova I, et al. Randomized, Phase III trial of panitumumab with infusional fluorouracil, leucovorin, and oxaliplatin (FOLFOX4) Versus FOLFOX4 alone as first-line treatment in patients with previously untreated metastatic colorectal cancer: The PRIME study. *J Clin Oncol* 2010;28:4697–705.

155. O’Shaughnessy J, Osborne C, Pippen J, Yoffe M, Patt D, Monaghan G, Rocha C, Ossovskaya V, Sherman B, Bradley C. Efficacy of BSI-201, a poly (ADP-ribose) polymerase-1 (PARP1) inhibitor, in combination with gemcitabine/carboplatin (G/C) in patients with metastatic triple-negative breast cancer (TNBC): results of a randomized phase II trial. *EJC Suppl* 2009;7:7.

156. O’Shaughnessy J, Osborne C, Pippen JE, Yoffe M, Patt D, Rocha C, Koo IC, Sherman BM, Bradley C. Iniparib plus Chemotherapy in Metastatic Triple-Negative Breast Cancer. *N Engl J Med [Internet]* 2011;364:205–14. Available from: http://www.nejm.org/doi/abs/10.1056/NEJMoa1011418

157. Duenas-Gonzalez A, Zarba JJ, Alcedo JC, Pattarunataporn P, Beslija S, Patel F, Casanova L, Barraclough H, Orlando M. A phase III study comparing concurrent gemcitabine (Gem) plus cisplatin (Cis) and radiation followed by adjuvant Gem plus Cis versus concurrent Cis and radiation in patients with stage IIB to IVA carcinoma of the cervix. *EJC Suppl* 2009;7:7.

158. Dueñas-González A, Zarbá JJ, Patel F, Alcedo JC, Beslija S, Casanova L, Pattaranutaporn P, Hameed S, Blair JM, Barraclough H, Orlando M. Phase III, open-label, randomized study comparing concurrent gemcitabine plus cisplatin and radiation followed by adjuvant gemcitabine and cisplatin versus concurrent cisplatin and radiation in patients with stage IIB to IVA carcinoma of the cervix. *J Clin Oncol* 2011;29:1678–85.

159. Van Cutsem E, Kang YK, Shen L, Lordick F, Ohtsu A, Satoh T, Hill J, Lehle M, Feyereislova A, Bang YJ. Trastuzumab added to standard chemotherapy (CT) as first-line treatment in human epidermal growth factor receptor 2 (HER2)-positive advanced gastric cancer (GC): efficacy and safety results from the Phase III ToGA trial. *EJC Suppl* 2009;7:7.

160. Bang YJ, Van Cutsem E, Feyereislova A, Chung HC, Shen L, Sawaki A, Lordick F, Ohtsu A, Omuro Y, Satoh T, Aprile G, Kulikov E, et al. Trastuzumab in combination with chemotherapy versus chemotherapy alone for treatment of HER2-positive advanced gastric or gastro-oesophageal junction cancer (ToGA): A phase 3, open-label, randomised controlled trial. *Lancet [Internet]* 2010;376:687–97. Available from: http://dx.doi.org/10.1016/S0140-6736(10)61121-X

161. Nutting C, A’Hern R, Rogers MS, Sydenham MA, Adab F, Harrington K, Jefferies S, Scrase C, Yap BK, Hall E, Grp PTM. First results of a phase III multicenter randomized controlled trial of intensity modulated (IMRT) versus conventional radiotherapy (RT) in head and neck cancer (PARSPORT: ISRCTN48243537; CRUK/03/005). *EJC Suppl* 2009;7:8.

162. Nutting CM, Morden JP, Harrington KJ, Urbano TG, Bhide SA, Clark C, Miles EA, Miah AB, Newbold K, Tanay M, Adab F, Jefferies SJ, et al. Parotid-sparing intensity modulated versus conventional radiotherapy in head and neck cancer (PARSPORT): a phase 3 multicentre randomised controlled trial. *Lancet Oncol* 2011;12:127–36.

163. Eggermont AMM, Suciu S, Testori A, Patel P, Spatz A, Grp EM. Ulceration of primary melanoma and responsiveness to adjuvant interferon therapy: Analysis of the adjuvant trials EORTC18952 and EORTC18991 in 2,644 patients. *EJC Suppl* 2009;7:8.

164. Eggermont AMM, Suciu S, Testori A, Kruit WH, Marsden J, Punt CJ, Santinami M, Sals F, Schadendorf D, Patel P, Dummer R, Robert C, et al. Ulceration and stage are predictive of interferon efficacy in melanoma: Results of the phase III adjuvant trials EORTC 18952 and EORTC 18991. *Eur J Cancer* 2012;48:218–25.

165. Kwak EL, Camidge DR, Clark J, Shapiro GI, Maki RG, Ratain MJ, Solomon B, Bang Y, Ou S, Salgia R. Clinical activity observed in a phase I dose escalation trial of an oral c-met and ALK inhibitor, PF-02341066. *EJC Suppl* 2009;7:8.

166. Miller VA, Hirsh V, Cadranel J, Chen Y-M, Park K, Kim S-W, Caicun Z, Oberdick M, Shahidi M, Yang C-H. PHASE IIB/III DOUBLE-BLIND RANDOMIZED TRIAL OF AFATINIB (BIBW 2992, AN IRREVERSIBLE INHIBITOR OF EGFR/HER1 AND HER2) + BEST SUPPORTIVE CARE (BSC) VERSUS PLACEBO plus BSC IN PATIENTS WITH NSCLC FAILING 1-2 LINES OF CHEMOTHERAPY AND ERLOTINIB OR GEFITINIB (. *Ann Oncol* 2010;21:1.

167. Miller VA, Hirsh V, Cadranel J, Chen YM, Park K, Kim SW, Zhou C, Su WC, Wang M, Sun Y, Heo DS, Crino L, et al. Afatinib versus placebo for patients with advanced, metastatic non-small-cell lung cancer after failure of erlotinib, gefitinib, or both, and one or two lines of chemotherapy (LUX-Lung 1): A phase 2b/3 randomised trial. *Lancet Oncol* 2012;13:528–38.

168. Yang C-H, Fukuoka M, Mok TS, Wu Y-L, Thongprasert S, Saijo N, Chu D-T, Jiang H, Duffield EL, Ichinose Y. FINAL OVERALL SURVIVAL (OS) RESULTS FROM A PHASE III, RANDOMISED, OPEN-LABEL, FIRST-LINE STUDY OF GEFITINIB (G) V CARBOPLATIN/PACLITAXEL (C/P) IN CLINICALLY SELECTED PATIENTS WITH ADVANCED NON-SMALL CELL LUNG CANCER (NSCLC) IN ASIA (IPASS). *Ann Oncol* 2010;21:1–2.

169. Fukuoka M, Wu YL, Thongprasert S, Sunpaweravong P, Leong SS, Sriuranpong V, Chao TY, Nakagawa K, Chu DT, Saijo N, Duffield EL, Rukazenkov Y, et al. Biomarker analyses and final overall survival results from a phase III, randomized, open-label, first-line study of gefitinib versus carboplatin/paclitaxel in clinically selected patients with advanced non - small-cell lung cancer in Asia (IPASS). *J Clin Oncol* 2011;29:2866–74.

170. Perez EA, Dirix L, Kocsis J, Gianni L, Lu J, Vinholes J, Ng V, Linehan C, Agresta S, Hurvitz S. EFFICACY AND SAFETY OF TRASTUZUMAB-DM1 VERSUS TRASTUZUMAB PLUS DOCETAXEL IN HER2-POSITIVE METASTATIC BREAST CANCER PATIENTS WITH NO PRIOR CHEMOTHERAPY FOR METASTATIC DISEASE: PRELIMINARY RESULTS OF A RANDOMIZED, MULTICENTER, OPEN-LABEL PHASE 2 STUDY (TDM4. *Ann Oncol* 2010;21:2.

171. Perez EA, Hurvitz SA, Amler LC, Mundt KE, Ng V, Guardino E, Gianni L. Relationship between HER2 expression and efficacy with first-line trastuzumab emtansine compared with trastuzumab plus docetaxel in TDM4450g: A randomized phase II study of patients with previously untreated HER2-positive metastatic breast cancer. *Breast Cancer Res* 2014;16:R50.

172. Perren T, Swart AM, Pfisterer J, Ledermann J, Lortholary A, Kristensen G, Carey M, Beale P, Cervantes A, Oza A, Collaborators GI. ICON7: A PHASE III RANDOMISED GYNAECOLOGIC CANCER INTERGROUP TRIAL OF CONCURRENT BEVACIZUMAB AND CHEMOTHERAPY FOLLOWED BY MAINTENANCE BEVACIZUMAB, VERSUS CHEMOTHERAPY ALONE IN WOMEN WITH NEWLY DIAGNOSED EPITHELIAL OVARIAN (EOC), PRIMARY PERITONEAL (PPC) O. *Ann Oncol* 2010;21:2–3.

173. Perren TJ, Swart AM, Pfisterer J, Ledermann JA, Pujade-Lauraine E, Kristensen G, Carey MS, Beale P, Cervantes A, Kurzeder C, Bois A du, Sehouli J, et al. A Phase 3 Trial of Bevacizumab in Ovarian Cancer. *N Engl J Med [Internet]* 2011;365:2484–96. Available from: http://www.nejm.org/doi/10.1056/NEJMoa1103799

174. de Bono JS, Logothetis CJ, Fizazi K, North S, Chu L, Chi KN, Kheoh T, Haqq C, Molina A, Scher HI, Investigators COUAA 301. ABIRATERONE ACETATE (AA) PLUS LOW DOSE PREDNISONE (P) IMPROVES OVERALL SURVIVAL (OS) IN PATIENTS (PTS) WITH METASTATIC CASTRATION-RESISTANT PROSTATE CANCER (MCRPC) WHO HAVE PROGRESSED AFTER DOCETAXEL-BASED CHEMOTHERAPY (CHEMO): RESULTS OF COU-AA-301, A RA. *Ann Oncol* 2010;21:3.

175. De Bono JS, Logothetis CJ, Molina A, Fizazi K, North S, Chu L, Chi KN, Jones RJ, Goodman Jr. OB, Saad F, Staffurth JN, Mainwaring P, et al. Abiraterone and Increased Survival in Metastatic Prostate Cancer. *N Engl J Med* 2011;364:1995–2005.

176. Dirix L, Migden MR, Oro AE, Hauschild A, Lewis K, Mueller AB, Yauch R, Reddy JC, Sekulic A. A Pivotal Multicenter Trial Evaluating Efficacy and Safety of the Hedgehog Pathway Inhibitor (HPI) Vismodegib in Patients With Advanced Basal Cell Carcinoma (BCC). *Eur J Cancer* 2011;47:2.

177. Sekulic A, Migden MR, Oro AE, Dirix L, Lewis KD, Hainsworth JD, Solomon JA, Yoo S, Arron ST, Friedlander PA, Marmur E, Rudin CM, et al. Efficacy and Safety of Vismodegib in Advanced Basal-Cell Carcinoma. *N Engl J Med [Internet]* 2012;366:2171–9. Available from: http://www.nejm.org/doi/abs/10.1056/NEJMoa1113713

178. Parker C, Heinrich D, O’Sullivan JM, Fossa S, Chodacki A, Demkow T, Cross A, Bolstad B, Garcia-Vargas J, Sartor O. Overall Survival Benefit of Radium-223 Chloride (Alpharadin (TM)) in the Treatment of Patients with Symptomatic Bone Metastases in Castration-resistant Prostate Cancer (CRPC): a Phase III Randomized Trial (ALSYMPCA). *Eur J Cancer* 2011;47:3.

179. Parker C, Nilsson S, Heinrich D, Helle SI, O’Sullivan JM, Fossa SD, Chodacki A, Wiechno P, Logue J, Seke M, Widmark A, Johannessen DC, et al. Alpha Emitter Radium-223 and Survival in Metastatic Prostate Cancer. *N Engl J Med* 2013;369:213–23.

180. Bourhis J, Sire C, Graff P, Gregoire V, Maingon P, Lapeyre M, Tortochaux JCG, Gery B, Martin L, Alfonsi M, Deprez P, Pignon T, et al. Very Accelerated Radiotherapy (RT) Versus Concomitant Chemo-Radiotherapy (Ct-Rt) In Locally Advanced Head And Neck Cancer: Long Term Results From 2 Phases III GORTEC Randomized Trials. *Eur J Cancer* 2011;47:4.

181. Bourhis J, Sire C, Graff P, Grégoire V, Maingon P, Calais G, Gery B, Martin L, Alfonsi M, Desprez P, Pignon T, Bardet E, et al. Concomitant chemoradiotherapy versus acceleration of radiotherapy with or without concomitant chemotherapy in locally advanced head and neck carcinoma (GORTEC 99-02): An open-label phase 3 randomised trial. *Lancet Oncol* 2012;13:145–53.

182. Bebin M, Franz DN, Sahmoud T, Belousova E, Sparagana S, Frost M, Ford J, Shah G, Cauwel H, Jozwiak S. Everolimus in Subependymal Giant Cell Astrocytomas (SEGA) Associated with Tuberous Sclerosis Complex (TSC): Results of EXIST-1, a Double-Blind Placebo-controlled Phase III Trial. *Eur J Cancer* 2011;47:4–5.

183. Franz DN, Belousova E, Sparagana S, Bebin EM, Frost M, Kuperman R, Witt O, Kohrman MH, Flamini JR, Wu JY, Curatolo P, De Vries PJ, et al. Efficacy and safety of everolimus for subependymal giant cell astrocytomas associated with tuberous sclerosis complex (EXIST-1): A multicentre, randomised, placebo-controlled phase 3 trial. *Lancet* 2013;381:125–32.

184. Fernando I, Bowden SJ, Brookes CL, Grieve R, Spooner D, Agrawal RK, Brunt AM, Churn M, Rea DW, Canney P. Synchronous Chemo-radiation Can Reduce Local Recurrence in Early Stage Breast Cancer: Results of the SECRAB Trial (ISRCTN: 84214355) Presented on Behalf of the SECRAB Steering Committee. *Eur J Cancer* 2011;47:2.

185. Tabernero J, Van Cutsem E, Lakomy R, Prausova J, Ruff P, Van Hazel G, Moiseyenko V, Ferry D, McKendrick J, Soussan-Lazard K, Boelle E, Allegra C. Results From VELOUR, a Phase 3 Study of Aflibercept (A) Versus Placebo (pbo) in Combination with FOLFIRI for the Treatment of Patients (pt) with Previously Treated Metastatic Colorectal Cancer (MCRC). *Eur J Cancer* 2011;47:5.

186. Tabernero J, Van Cutsem E, Lakomý R, Prausová J, Ruff P, Van Hazel GA, Moiseyenko VM, Ferry DR, McKendrick JJ, Soussan-Lazard K, Chevalier S, Allegra CJ. Aflibercept versus placebo in combination with fluorouracil, leucovorin and irinotecan in the treatment of previously treated metastatic colorectal cancer: Prespecified subgroup analyses from the VELOUR trial. *Eur J Cancer* 2014;50:320–31.

187. Aghajanian C, Blank S V, Goff B, Judson PL, Makhija S, Sharma SK, Nycum LR, Yi J, Husain A. Efficacy in Patient Subgroups in OCEANS, a Randomized, Double-blinded, Placebo-controlled, Phase 3 Trial of Chemotherapy +/- Bevacizumab in Patients with Platinum-sensitive Recurrent Epithelial Ovarian (OC), Primary Peritoneal (PPC), or Fallopian Tube Can. *Eur J Cancer* 2011;47:5.

188. Aghajanian C, Blank S V., Goff BA, Judson PL, Teneriello MG, Husain A, Sovak MA, Yi J, Nycum LR. OCEANS: A randomized, double-blind, placebo-controlled phase III trial of chemotherapy with or without bevacizumab in patients with platinum-sensitive recurrent epithelial ovarian, primary peritoneal, or fallopian tube cancer. *J Clin Oncol* 2012;30:2039–45.

189. Hoskin P, Sundar S, Reczko K, Forsyth S, Mithal N, Sizer B, Toy L, Stratford M, Jitlal M. A Multicentre Randomised Trial of Ibandronate Compared to Single Dose Radiotherapy for Localised Metastatic Bone Pain in Prostate Cancer (RIB). *Eur J Cancer* 2011;47:6.

190. Hoskin P, Sundar S, Reczko K, Forsyth S, Mithal N, Sizer B, Bloomfield D, Upadhyay S, Wilson P, Kirkwood A, Stratford M, Jitlal M, et al. A Multicenter Randomized Trial of Ibandronate Compared with Single-Dose Radiotherapy for Localized Metastatic Bone Pain in Prostate Cancer. *J Natl Cancer Inst* 2015;107.

191. Sullivan R, Peppercorn J, Sikora K, Zalcberg J, Meropol N, Amir E, Khayat D, Boyle P, Tannock I, Fojo T. Delivering Affordable Cancer Care in High-income Countries: a Lancet Oncology Commission. *Eur J Cancer* 2011;47:6.

192. Sullivan R, Peppercorn J, Sikora K, Zalcberg J, Meropol NJ, Amir E, Khayat D, Boyle P, Autier P, Tannock IF, Fojo T, Siderov J, et al. Delivering affordable cancer care in high-income countries. *Lancet Oncol* 2011;12:933–80.

193. Krug LM, Kindler H, Calvert H, Manegold C, Tsao AS, Fennell D, Lubiniecki GM, Sun X, Smith M, Baas P. VANTAGE 014: Vorinostat (V) in Patients With Advanced Malignant Pleural Mesothelioma (MPM) who Have Failed Prior Pemetrexed and Either Cisplatin or Carboplatin Therapy: A Phase iii, Randomized, Double-Blind, Placebo-Controlled Trial. *Eur J Cancer* 2011;47:2–3.

194. Krug LM, Kindler HL, Calvert H, Manegold C, Tsao AS, Fennell D, Öhman R, Plummer R, Eberhardt WEE, Fukuoka K, Gaafar RM, Lafitte JJ, et al. Vorinostat in patients with advanced malignant pleural mesothelioma who have progressed on previous chemotherapy (VANTAGE-014): A phase 3, double-blind, randomised, placebo-controlled trial. *Lancet Oncol* 2015;16:447–56.

195. Baselga J, Campone M, Sahmoud T, Piccart M, Burris H, Rugo H, Noguchi S, Gnant M, Mukhopadhyay P, Hortobagyi G. Everolimus in Combination with Exemestane for Postmenopausal Women with Advanced Breast Cancer Who Are Refractory to Letrozole or Anastrozole: Results of the BOLERO-2 Phase III Trial. *Eur J Cancer* 2011;47:6–7.

196. Piccart M, Hortobagyi GN, Campone M, Pritchard KI, Lebrun F, Ito Y, Noguchi S, Perez A, Rugo HS, Deleu I, Burris HA, Provencher L, et al. Everolimus plus exemestane for hormonereceptor- positive, human epidermal growth factor receptor-2-negative advanced breast cancer: Overall survival results from BOLERO-2. *Ann Oncol* 2014;25:2357–62.

197. Piccart M, Bogaerts J, Cardoso F, Werutsky G, Delaloge S, Van’t Veer L, Rubio I, Moulin C, Engelen K, Viale G, Thompson AM, Passalacqua R, et al. The EORTC 10041/BIG 03-04 MINDACT (Microarray in Node Negative and 1 to 3 Positive Lymph Node Disease May Avoid ChemoTherapy) Trial: Patients’ Baseline Characteristics and Logistics Aspects After a Successful Accrual. *Eur J Cancer* 2011;47:7.

198. Rutgers E, Piccart-Gebhart MJ, Bogaerts J, Delaloge S, Veer LV t. L, Rubio IT, Viale G, Thompson AM, Passalacqua R, Nitz U, Vindevoghel A, Pierga JY, et al. The EORTC 10041/BIG 03-04 MINDACT trial is feasible: Results of the pilot phase. *Eur J Cancer* 2011;47:2742–9.

199. Bonjer HJ, Haglind E, Cuesta MA, Fuerst A, Lacy A, van der Pas MHGM, Hop WCJ, Grp CIIS. Laparoscopic Surgery Versus Open Surgery for Rectal Cancer: Short-term Outcomes of a Randomised Trial. *Eur J Cancer* 2011;47:4.

200. Bonjer HJ, Deijen CL, Abis GA, Cuesta MA, van der Pas MHGM, de Lange-de Klerk ESM, Lacy AM, Bemelman WA, Andersson J, Angenete E, Rosenberg J, Fuerst A, et al. A Randomized Trial of Laparoscopic versus Open Surgery for Rectal Cancer. *N Engl J Med [Internet]* 2015;372:1324–32. Available from: http://www.nejm.org/doi/10.1056/NEJMoa1414882

201. Van Hemelrijck M, Stocks T, Jonsson H, Manjer J, Ulmer H, Bjorge T, Stattin P. Blood Pressure and Risk of Incident and Fatal Cancer in the Metabolic Syndrome and Cancer Project (Me-Can) - Analysis of Seven Prospective Cohorts. *Eur J Cancer* 2011;47:3.

202. Stocks T, Van Hemelrijck M, Manjer J, Bjørge T, Ulmer H, Hallmans G, Lindkvist B, Selmer R, Nagel G, Tretli S, Concin H, Engeland A, et al. Blood pressure and risk of cancer incidence and mortality in the metabolic syndrome and cancer project. *Hypertension* 2012;59:802–10.

203. Amant F, Van Calsteren K, Halaska M, Mhallem M, Lagae L, Willemsen M, Kapusta L, Van Calster B, Mertens L, Ottevanger P. Cognitive and Cardiac Outcome After Prenatal Exposure to Chemotherapy in Children 18 Months or Older. *Eur J Cancer* 2011;47:8.

204. Amant F, Van Calsteren K, Halaska MJ, Gziri MM, Hui W, Lagae L, Willemsen MA, Kapusta L, Van Calster B, Wouters H, Heyns L, Han SN, et al. Long-term cognitive and cardiac outcomes after prenatal exposure to chemotherapy in children aged 18 months or older: An observational study. *Lancet Oncol* 2012;13:256–64.

205. Papaemmanuil E, Malcovati L, Cazzola M, Hellstrom-Lindberg E, Bowen D, Boultwood JB, Green AR, Futreal PA, Stratton MR, Campbell PJ. Identification of Novel Somatic Mutations in SF3B1, a Gene Encoding a Core Component of RNA Splicing Machinery, in Myelodysplasia with Ring Sideroblasts and Other Common Cancers. *Eur J Cancer* 2011;47:7.

206. Papaemmanuil E, Cazzola M, Boultwood J, Malcovati L, Vyas P, Bowen D, Pellagatti A, Wainscoat JS, Hellstrom-Lindberg E, Gambacorti-Passerini C, Godfrey AL, Rapado I, et al. Somatic *SF3B1* Mutation in Myelodysplasia with Ring Sideroblasts. *N Engl J Med [Internet]* 2011;365:1384–95. Available from: http://www.nejm.org/doi/abs/10.1056/NEJMoa1103283

207. Middleton M, Friedlander P, Hamid O, Daud A, Plummer R, Schuster R, Qian J, Luo Y, Giranda V, McArthur G. Efficacy of Veliparib (ABT-888) Plus Temozolomide Versus Temozolomide Alone: a Randomized, Double-blind, Placebo-controlled Trial in Patients with Metastatic Melanoma. *Eur J Cancer* 2011;47:8.

208. Middleton MR, Friedlander P, Hamid O, Daud A, Plummer R, Falotico N, Chyla B, Jiang F, McKeegan E, Mostafa NM, Zhu M, Qian J, et al. Randomized phase II study evaluating veliparib (ABT-888) with temozolomide in patients with metastatic melanoma. *Ann Oncol* 2015;26:2173–9.

209. Van Cutsem E, Prenen H, Guillen-Ponce C, Bennouna J, Di Benedetto M, Bouche O, Staines H, Oum’Hamed Z, Studeny M, Capdevila J. A Phase I/II, Open-label, Randomised Study of BIBF 1120 Plus mFOLFOX6 Compared to Bevacizumab Plus mFOLFOX6 in Patients with Metastatic Colorectal Cancer. *Eur J Cancer* 2011;47:8–9.

210. Van Cutsem E, Prenen H, D’Haens G, Bennouna J, Carrato A, Ducreux M, Bouché O, Sobrero A, Latini L, Staines H, Oum’Hamed Z, Dressler H, et al. A phase I/II, open-label, randomised study of nintedanib plus mFOLFOX6 versus bevacizumab plus mFOLFOX6 in first-line metastatic colorectal cancer patients. *Ann Oncol* 2015;26:2085–91.

211. Shaw AT, Kim DW, Nakagawa K, Seto T, Crino L, Ahn M, De Pas T, Besse B, Solomon B, Blackhall FH, Wu Y, Thomas M, et al. PHASE III STUDY OF CRIZOTINIB VERSUS PEMETREXED OR DOCETAXEL CHEMOTHERAPY IN PATIENTS WITH ADVANCED ALK-POSITIVE NON-SMALL CELL LUNG CANCER (NSCLC) (PROFILE 1007). *Ann Oncol [Internet]* 2012;23:LBA1. Available from: https://dx.doi.org/10.1093/annonc/mds499

212. Shaw AT, Kim D-W, Nakagawa K, Seto T, Crinó L, Ahn M-J, De Pas T, Besse B, Solomon BJ, Blackhall F, Wu Y-L, Thomas M, et al. Crizotinib versus Chemotherapy in Advanced *ALK* -Positive Lung Cancer. *N Engl J Med [Internet]* 2013;368:2385–94. Available from: http://www.nejm.org/doi/10.1056/NEJMoa1214886

213. Zhu AX, Rosmorduc O, Evans J, Ross P, Santoro A, Carrilho FJ, Leberre M, Jensen M, Meinhardt G, Kang Y. A PHASE III, RANDOMIZED, DOUBLE-BLIND, PLACEBO-CONTROLLED TRIAL OF SORAFENIB PLUS ERLOTINIB IN PATIENTS WITH HEPATOCELLULAR CARCINOMA (HCC). *Ann Oncol [Internet]* 2012;23:LBA2. Available from: https://dx.doi.org/10.1093/annonc/mds499

214. Zhu AX, Rosmorduc O, Evans TRJ, Ross PJ, Santoro A, Carrilho FJ, Bruix J, Qin S, Thuluvath PJ, Llovet JM, Leberre MA, Jensen M, et al. Search: A phase III, randomized, double-blind, placebo-controlled trial of sorafenib plus erlotinib in patients with advanced hepatocellular carcinoma. *J Clin Oncol* 2015;33:559–66.

215. Lordick F, Bedoky G, Chung H, Kurteva G, Kang Y, Oh SC, Salman P, Goette H, Melezinkova H, Moehler M. CETUXIMAB IN COMBINATION WITH CAPECITABINE AND CISPLATIN AS FIRST-LINE TREATMENT IN ADVANCED GASTRIC CANCER: RANDOMIZED CONTROLLED PHASE III EXPAND STUDY. *Ann Oncol [Internet]* 2012;23:LBA3. Available from: https://dx.doi.org/10.1093/annonc/mds499

216. Lordick F, Kang YK, Chung HC, Salman P, Oh SC, Bodoky G, Kurteva G, Volovat C, Moiseyenko VM, Gorbunova V, Park JO, Sawaki A, et al. Capecitabine and cisplatin with or without cetuximab for patients with previously untreated advanced gastric cancer (EXPAND): A randomised, open-label phase 3 trial. *Lancet Oncol* 2013;14:490–9.

217. Taieb J, Tabernero J, Mini E, Subtil F, Folprecht G, Van Laethem J-L, Thaler J, Bridgewater J, Van Cutsem E, Rougier P, Collette L, Praet M, et al. ADJUVANT FOLFOX4 WITH OR WITHOUT CETUXIMAB (CTX) IN PATIENTS (PTS) WITH RESECTED STAGE III COLON CANCER (CC): DFS AND OS RESULTS AND SUBGROUP ANALYSES OF THE PETACC8 INTERGROUP PHASE III TRIAL. *Ann Oncol [Internet]* 2012;23:LBA4. Available from: https://dx.doi.org/10.1093/annonc/mds499

218. Taieb J, Tabernero J, Mini E, Subtil F, Folprecht G, Van Laethem JL, Thaler J, Bridgewater J, Petersen LN, Blons H, Collette L, Van Cutsem E, et al. Oxaliplatin, fluorouracil, and leucovorin with or without cetuximab in patients with resected stage III colon cancer (PETACC-8): An open-label, randomised phase 3 trial. *Lancet Oncol* 2014;15:862–73.

219. Pivot X, Romieu G, Bonnefoi H, Pierga J-Y, Kerbrat P, Guastalla J-P, Lortholary A, Espie M, Fumoleau P, Khayat D, Pauporte I, Kramar A. PHARE Trial results of subset analysis comparing 6 to 12 months of trastuzumab in adjuvant early breast cancer. *Ann Oncol* 2012;23:LBA5.

220. Pivot X, Romieu G, Debled M, Pierga JY, Kerbrat P, Bachelot T, Lortholary A, Espié M, Fumoleau P, Serin D, Jacquin JP, Jouannaud C, et al. 6 months versus 12 months of adjuvant trastuzumab for patients with HER2-positive early breast cancer (PHARE): A randomised phase 3 trial. *Lancet Oncol* 2013;14:741–8.

221. Goldhirsch A, Piccart M, Procter M, De Azambuja E, Weber H, Untch M, Smith IE, Gianni L, Jackisch C, Cameron D, Bell R, Dowsett M, et al. HERA TRIAL: 2 years versus 1 year of trastuzumab after adjuvant chemotherapy in women with HER2-positive early breast cancer at 8 years of median follow up. *Ann Oncol [Internet]* 2012;23:LBA6. Available from: https://dx.doi.org/10.1093/annonc/mds499

222. Goldhirsch A, Gelber RD, Piccart-Gebhart MJ, De Azambuja E, Procter M, Suter TM, Jackisch C, Cameron D, Weber HA, Heinzmann D, Lago LD, McFadden E, et al. 2 years versus 1 year of adjuvant trastuzumab for HER2-positive breast cancer (HERA): An open-label, randomised controlled trial. *Lancet* 2013;382:1021–8.

223. Judson I, Verweij J, Gelderblom H, Hartmann JT, Schöffski P, Blay J, Dei Tos AP, Marreaud S, Litiere S, Van der Graaf WTA. RESULTS OF A RANDOMISED PHASE III TRIAL (EORTC 62012) OF SINGLE AGENT DOXORUBICIN VERSUS DOXORUBICIN PLUS IFOSFAMIDE AS FIRST LINE CHEMOTHERAPY FOR PATIENTS WITH ADVANCED OR METASTATIC SOFT TISSUE SARCOMA: A SURVIVAL STUDY BY THE EORTC SOFT TISSUE AND BON. *Ann Oncol [Internet]* 2012;23:LBA7. Available from: https://dx.doi.org/10.1093/annonc/mds499

224. Judson I, Verweij J, Gelderblom H, Hartmann JT, Schöffski P, Blay JY, Kerst JM, Sufliarsky J, Whelan J, Hohenberger P, Krarup-Hansen A, Alcindor T, et al. Doxorubicin alone versus intensified doxorubicin plus ifosfamide for first-line treatment of advanced or metastatic soft-tissue sarcoma: A randomised controlled phase 3 trial. *Lancet Oncol* 2014;15:415–23.

225. Motzer R j, Hutson TE, Reeves J, Hawkins R, Guo J, Nathan P, Staehler M, de Souza P, Merchan JR, Fife K, Jin J, Jones R, et al. RANDOMIZED, OPEN-LABEL, PHASE III TRIAL OF PAZOPANIB VERSUS SUNITINIB IN FIRST-LINE TREATMENT OF PATIENTS WITH METASTATIC RENAL CELL CARCINOMA (MRCC): RESULTS OF THE COMPARZ TRIAL. *Ann Oncol [Internet]* 2012;23:LBA8. Available from: https://dx.doi.org/10.1093/annonc/mds499

226. Motzer RJ, Hutson TE, Cella D, Reeves J, Hawkins R, Guo J, Nathan P, Staehler M, de Souza P, Merchan JR, Boleti E, Fife K, et al. Pazopanib versus Sunitinib in Metastatic Renal-Cell Carcinoma. *N Engl J Med* 2013;369:722–31.

227. Autier P, Boniol M, Pizot C, Mullie P. Vitamin D status and inflammation in cancer and other diseases. *Eur J Cancer* 2013;49:S2.

228. Autier P, Boniol M, Pizot C, Mullie P. Vitamin D status and ill health: A systematic review. *Lancet Diabetes Endocrinol* 2014;2:76–89.

229. Poortmans P, Struikmans H, Kirkove C, Budach V, Maingon P, Valli MC, Collette S, Fourquet A, Bartelink H, den Bogaert W. Irradiation of the internal mammary and medial supraclavicular lymph nodes in stage I to III breast cancer: 10 years results of the EORTC Radiation Oncology and Breast Cancer Groups phase III trial 22922/10925. *Eur J Cancer* 2013;49:S1–2.

230. Poortmans PM, Collette S, Kirkove C, Van Limbergen E, Budach V, Struikmans H, Collette L, Fourquet A, Maingon P, Valli M, De Winter K, Marnitz S, et al. Internal Mammary and Medial Supraclavicular Irradiation in Breast Cancer. *N Engl J Med [Internet]* 2015;373:317–27. Available from: http://www.nejm.org/doi/10.1056/NEJMoa1415369

231. Breugom AJ, van den Broek CBM, van Gijn W, Putter H, Kranenbarg EM-K, Glimelius B, Pahlman L, Rutten HJT, Marijnen CAM, van de Velde CJH, Grp DCC. The value of adjuvant chemotherapy in rectal cancer patients after preoperative radiotherapy or chemoradiation followed by TME-surgery: The PROCTOR/SCRIPT study. *Eur J Cancer* 2013;49:S1.

232. Breugom AJ, Swets M, Bosset JF, Collette L, Sainato A, Cionini L, Glynne-Jones R, Counsell N, Bastiaannet E, van den Broek CBM, Liefers GJ, Putter H, et al. Adjuvant chemotherapy after preoperative (chemo)radiotherapy and surgery for patients with rectal cancer: A systematic review and meta-analysis of individual patient data. *Lancet Oncol* 2015;16:200–7.

233. Reimers MS, Kuppen PJK, Lee M, Tezcan H, Putter H, Lopatin M, Clark-Langone K, Liefers GJ, Shak S, van de Velde CJH. Validation of the 12-gene colon cancer recurrence score (R) assay as a predictor of recurrence risk in stage II and III rectal cancer patients. *Eur J Cancer* 2013;49:S2.

234. Reimers MS, Kuppen PJK, Lee M, Lopatin M, Tezcan H, Putter H, Clark-Langone K, Liefers GJ a., Shak S, van de Velde CJH. Validation of the 12-gene colon cancer recurrence score as a predictor of recurrence risk in stage II and III rectal cancer patients. *J Natl Cancer Inst* 2014;106.

235. Giaccone G, Bazhenova L, Nemunaitis J, Juhasz E, Ramlau R, van den Heuvel MM, Lal R, Dunlop DJ, Carrier E, Fakhrai H. A phase III study of belagenpumatucel-L therapeutic tumor cell vaccine for non-small cell lung cancer (NSCLC). *Eur J Cancer* 2013;49:S2–3.

236. Giaccone G, Bazhenova LA, Nemunaitis J, Tan M, Juhász E, Ramlau R, Van Den Heuvel MM, Lal R, Kloecker GH, Eaton KD, Chu Q, Dunlop DJ, et al. A phase III study of belagenpumatucel-L, an allogeneic tumour cell vaccine, as maintenance therapy for non-small cell lung cancer. *Eur J Cancer* 2015;51:2321–9.

237. Caplin M, Ruszniewski P, Pavel M, Cwikla J, Phan A, Raderer M, Sedlackova E, Cadiot G, Wall L, Rindi G, Liyanage N, Blumberg J. A randomized, double-blind, placebo-Controlled study of Lanreotide Antiproliferative Response in patients with gastroenteropancreatic NeuroEndocrine Tumors (CLARINET). *Eur J Cancer* 2013;49:S3.

238. Caplin ME, Pavel M, Ćwikła JB, Phan AT, Raderer M, Sedláčková E, Cadiot G, Wolin EM, Capdevila J, Wall L, Rindi G, Langley A, et al. Lanreotide in Metastatic Enteropancreatic Neuroendocrine Tumors. *N Engl J Med [Internet]* 2014;371:224–33. Available from: http://www.nejm.org/doi/10.1056/NEJMoa1316158

239. Brastianos PK, Carter SL, Santagata S, Taylor-Weiner A, Stemmer-Rachamimov A, Louis DL, Baselga J, Beroukhim R, Getz G, Hahn WC. Genomic characterization of brain metastases and paired primary tumors reveals patterns of clonal evolution. *Eur J Cancer* 2013;49:S3.

240. Brastianos PK, Carter SL, Santagata S, Cahill DP, Taylor-Weiner A, Jones RT, Van Allen EM, Lawrence MS, Horowitz PM, Cibulskis K, Ligon KL, Tabernero J, et al. Genomic characterization of brain metastases reveals branched evolution and potential therapeutic targets. *Cancer Discov* 2015;5:1164–77.

241. Witteveen P, Lortholary A, Fehm T, Poveda A, Reuss A, Havsteen H, Raspagliesi F, Vergote I, Bamias A, Pujade-Lauraine E. Final overall survival (OS) results from AURELIA, an open-label randomised phase III trial of chemotherapy (CT) with or without bevacizumab (BEV) for platinum-resistant recurrent ovarian cancer (OC). *Eur J Cancer* 2013;49:S3–4.

242. Pujade-Lauraine E, Hilpert F, Weber B, Reuss A, Poveda A, Kristensen G, Sorio R, Vergote I, Witteveen P, Bamias A, Pereira D, Wimberger P, et al. Bevacizumab combined with chemotherapy for platinum-resistant recurrent ovarian cancer: The AURELIA open-label randomized phase III trial. *J Clin Oncol* 2014;32:1302–8.

243. Oza AM, Perren TJ, Swart AM, Schroeder W, Pujade-Lauraine E, Havsteen H, Beale P, Cervantes AM, Embleton AC, Parmar M. ICON7: Final overall survival results in the GCIG phase III randomized trial of bevacizumab in women with newly diagnosed ovarian cancer. *Eur J Cancer* 2013;49:S4.

244. Oza AM, Cook AD, Pfisterer J, Embleton A, Ledermann JA, Pujade-Lauraine E, Kristensen G, Carey MS, Beale P, Cervantes A, Park-Simon TW, Rustin G, et al. Standard chemotherapy with or without bevacizumab for women with newly diagnosed ovarian cancer (ICON7): Overall survival results of a phase 3 randomised trial. *Lancet Oncol* 2015;16:928–36.

245. Sclafani F, Gonzalez D, Cunningham D, Wilson SH, Peckitt C, Tabernero J, Glimelius B, Cervantes A, Brown G, Chau I. TP53 status may predict benefit from cetuximab in high-risk, locally advanced rectal cancer: Results of the EXPERT-C trial. *Eur J Cancer* 2013;49:S4.

246. Sclafani F, Gonzalez D, Cunningham D, Hulkki Wilson S, Peckitt C, Giralt J, Glimelius B, Roselló Keränen S, Wotherspoon A, Brown G, Tait D, Oates J, et al. RAS mutations and cetuximab in locally advanced rectal cancer: Results of the EXPERT-C trial. *Eur J Cancer* 2014;50:1430–6.

247. Paoletti X, Le Tourneau C, Collette L, Rizzo E, Lacombe D, Olmos D, Siu L, Percy Y, Seymour L, Kaye SB, Verweij J, Massard C, et al. Towards new methods for the determination of dose limiting toxicities and the assessment of the recommended dose for further studies of non-cytotoxic agents. *Eur J Cancer* 2013;49:S5.

248. Postel-Vinay S, Collette L, Paoletti X, Rizzo E, Massard C, Olmos D, Fowst C, Levy B, Mancini P, Lacombe D, Ivy P, Seymour L, et al. Towards new methods for the determination of dose limiting toxicities and the assessment of the recommended dose for further studies of molecularly targeted agents - Dose-Limiting Toxicity and Toxicity Assessment Recommendation Group for Early Trials of T. *Eur J Cancer* 2014;50:2040–9.

249. Coleman R, Hinsley S, Bell R, Cameron D, Dodwell D, Liversedge V, Burkinshaw R, Keane M, Gil M, Marshall H. Adjuvant therapy in early breast cancer with zoledronic acid (AZURE - BIG 01/04): Final efficacy analysis. *Eur J Cancer* 2013;49:S5.

250. Coleman R, Cameron D, Dodwell D, Bell R, Wilson C, Rathbone E, Keane M, Gil M, Burkinshaw R, Grieve R, Barrett-Lee P, Ritchie D, et al. Adjuvant zoledronic acid in patients with early breast cancer: Final efficacy analysis of the AZURE (BIG 01/04) randomised open-label phase 3 trial. *Lancet Oncol* 2014;15:997–1006.

251. Ledermann JA, Perren TJ, Raja FA, Embleton A, Rustin GJS, Jayson G, Kaye SB, Swart AM, Vaughan M, Hirte H, Collaborators I. Randomised double-blind phase III trial of cediranib (AZD 2171) in relapsed platinum sensitive ovarian cancer: Results of the ICON6 trial. *Eur J Cancer* 2013;49:S5–6.

252. Ledermann JA, Embleton AC, Raja F, Perren TJ, Jayson GC, Rustin GJS, Kaye SB, Hirte H, Eisenhauer E, Vaughan M, Friedlander M, González-Martín A, et al. Cediranib in patients with relapsed platinum-sensitive ovarian cancer (ICON6): A randomised, double-blind, placebo-controlled phase 3 trial. *Lancet* 2016;387:1066–74.

253. Cheng J, Vollan HKM, Wedge DC, Borresen-Dale AL, Campbell PJ, Kristensen VN, Stratton MR, Lingjaerde OC, Moreau Y, Van Loo P. The landscape of tumor suppressors across human cancers. *Eur J Cancer* 2013;49:S6.

254. Cheng J, Demeulemeester J, Wedge DC, Vollan HKM, Pitt JJ, Russnes HG, Pandey BP, Nilsen G, Nord S, Bignell GR, White KP, Børresen-Dale AL, et al. Pan-cancer analysis of homozygous deletions in primary tumours uncovers rare tumour suppressors. *Nat Commun* 2017;8.

255. Eriksen JG, Maare C, Johansen J, Primdahl H, Evensen J, Kristensen CA, Andersen LJ, Overgaard J. DAHANCA 19: First results of a randomized phase III study of the importance of the EGFR-inhibitor zalutumumab for the outcome of primary curative radiotherapy for squamous cell carcinoma of the head and neck. *Eur J Cancer [Internet]* 2013;49:S6. Available from: http://www.embase.com/search/results?subaction=viewrecord&from=export&id=L71273039%5Cnhttp://dx.doi.org/10.1016/S0959-8049(13)70069-8

256. Chlebowski R, Anderson GL, Sarto G, Haque R, Runowicz C, Aragaki A, Thomson CA, Howard B V, Reed S, Manson JE. Continuous combined oestrogen plus progestin and endometrial cancer in postmenopausal women in the Women’s Health Initiative randomized clinical trial. *Eur J Cancer* 2013;49:S7.

257. Chlebowski RT, Anderson GL, Sarto GE, Haque R, Runowicz CD, Aragaki AK, Thomson CA, Howard B V., Wactawski-Wende J, Chen C, Rohan TE, Simon MS, et al. Continuous Combined Estrogen Plus Progestin and Endometrial Cancer: The Women’s Health Initiative Randomized Trial. *J Natl Cancer Inst* 2016;108.

258. de Koning HJ, Plevritis SK, Meza R, ten Haaf K, Munshi VN, Pinsky PF, Hazelton WD, Feuer EJ, McMahon PM, Grp CLM. Benefits and harms of computed tomography lung cancer screening programs for high risk populations - using evidence from the 2 largest randomized controlled trials on lung cancer screening worldwide. *Eur J Cancer* 2013;49:S7.

259. De Koning HJ, Meza R, Plevritis SK, Ten Haaf K, Munshi VN, Jeon J, Erdogan SA, Kong CY, Han SS, Van Rosmalen J, Choi SE, Pinsky PF, et al. Benefits and harms of computed tomography lung cancer screening strategies: A comparative modeling study for the U.S. Preventive services task force. *Ann Intern Med* 2014;160:311–20.

260. Weber JS, Minor DR, D’Angelo SP, Hodi FS, Gutzmer R, Neyns B, Hoeller C, Khushalani NI, Miller WH, Grob J-J, Lao C, Linette G, et al. A PHASE 3 RANDOMIZED, OPEN-LABEL STUDY OF NIVOLUMAB (ANTI-PD-1; BMS-936558; ONO-4538) VERSUS INVESTIGATOR’S CHOICE CHEMOTHERAPY (ICC) IN PATIENTS WITH ADVANCED MELANOMA AFTER PRIOR ANTI-CTLA-4 THERAPY. *Ann Oncol* 2014;25:LBA3.

261. Weber JS, D’Angelo SP, Minor D, Hodi FS, Gutzmer R, Neyns B, Hoeller C, Khushalani NI, Miller WH, Lao CD, Linette GP, Thomas L, et al. Nivolumab versus chemotherapy in patients with advanced melanoma who progressed after anti-CTLA-4 treatment (CheckMate 037): A randomised, controlled, open-label, phase 3 trial. *Lancet Oncol* 2015;16:375–84.

262. Robert C, Mortier L, Chiarion-Sileni V, Drucis K, Krajsova I, Hauschild A, Legos J, Sun P, Rubin SD, Little SM, Crist WA, Schadendorf D, et al. COMBI-V: A RANDOMISED, OPEN-LABEL, PHASE III STUDY COMPARING THE COMBINATION OF DABRAFENIB (D) AND TRAMETINIB (T) WITH VEMURAFENIB (V) AS FIRST-LINE THERAPY IN PATIENTS (PTS) WITH UNRESECTABLE OR METASTATIC BRAF V600E/K MUTATION-POSITIVE CUTANEOUS MELANOM. *Ann Oncol [Internet]* 2014;25:LBA4. Available from: https://dx.doi.org/10.1093/annonc/mdu438.39

263. Grob JJ, Amonkar MM, Karaszewska B, Schachter J, Dummer R, Mackiewicz A, Stroyakovskiy D, Drucis K, Grange F, Chiarion-Sileni V, Rutkowski P, Lichinitser M, et al. Comparison of dabrafenib and trametinib combination therapy with vemurafenib monotherapy on health-related quality of life in patients with unresectable or metastatic cutaneous BRAF Val600-mutation-positive melanoma (COMBI-v): Results of a phase 3, open-l. *Lancet Oncol* 2015;16:1389–98.

264. McArthur GA, Thomas L, De La Cruz Merino L, Atkinson V, Dutriaux C, Garbe C, Chang I, Hack SP, Dréno B, Ascierto PA, Larkin J, Ribas A, et al. PHASE 3, DOUBLE-BLIND, PLACEBO-CONTROLLED STUDY OF VEMURAFENIB VERSUS VEMURAFENIB + COBIMETINIB IN PREVIOUSLY UNTREATED BRAFV600 MUTATION–POSITIVE PATIENTS WITH UNRESECTABLE LOCALLY ADVANCED OR METASTATIC MELANOMA (NCT01689519). *Ann Oncol [Internet]* 2014;25:LBA5. Available from: https://dx.doi.org/10.1093/annonc/mdu438.40

265. Ascierto PA, McArthur GA, Dréno B, Atkinson V, Liszkay G, Di Giacomo AM, Mandalà M, Demidov L, Stroyakovskiy D, Thomas L, de la Cruz-Merino L, Dutriaux C, et al. Cobimetinib combined with vemurafenib in advanced BRAFV600-mutant melanoma (coBRIM): updated efficacy results from a randomised, double-blind, phase 3 trial. *Lancet Oncol* 2016;17:1248–60.

266. Swain S, Clark E, Ross G, Heeson S, Benyunes MC, Baselga J, Kim S, Cortes J, Ro J, Semiglazov V, Campone M, Ciruelos E, et al. 350O_PRFINAL OVERALL SURVIVAL (OS) ANALYSIS FROM THE CLEOPATRA STUDY OF FIRST-LINE (1L) PERTUZUMAB (PTZ), TRASTUZUMAB (T), AND DOCETAXEL (D) IN PATIENTS (PTS) WITH HER2-POSITIVE METASTATIC BREAST CANCER (MBC). *Ann Oncol [Internet]* 2014;25. Available from: https://dx.doi.org/10.1093/annonc/mdu438.7

267. Swain SM, Baselga J, Kim S-B, Ro J, Semiglazov V, Campone M, Ciruelos E, Ferrero J-M, Schneeweiss A, Heeson S, Clark E, Ross G, et al. Pertuzumab, Trastuzumab, and Docetaxel in HER2-Positive Metastatic Breast Cancer. *N Engl J Med [Internet]* 2015;372:724–34. Available from: http://www.nejm.org/doi/10.1056/NEJMoa1413513

268. Vansteenkiste JF, Nakayama H, Havel L, Kondo H, Mitsudomi T, Zarogoulidis K, Gladkov OA, Spiessens B, Debruyne C, Brichard V, Therasse P, Altorki N, et al. 1173OMAGRIT, A DOUBLE-BLIND, RANDOMIZED, PLACEBO-CONTROLLED PHASE III STUDY TO ASSESS THE EFFICACY OF THE RECMAGE-A3 + AS15 CANCER IMMUNOTHERAPEUTIC AS ADJUVANT THERAPY IN PATIENTS WITH RESECTED MAGE-A3-POSITIVE NON-SMALL CELL LUNG CANCER (NSCLC). *Ann Oncol [Internet]* 2014;25:iv409–iv409. Available from: https://dx.doi.org/10.1093/annonc/mdu347.1

269. Vansteenkiste JF, Cho BC, Vanakesa T, De Pas T, Zielinski M, Kim MS, Jassem J, Yoshimura M, Dahabreh J, Nakayama H, Havel L, Kondo H, et al. Efficacy of the MAGE-A3 cancer immunotherapeutic as adjuvant therapy in patients with resected MAGE-A3-positive non-small-cell lung cancer (MAGRIT): a randomised, double-blind, placebo-controlled, phase 3 trial. *Lancet Oncol* 2016;17:822–35.

270. Mok TSK, Atagi S, Ponce S, Jiang H, Shi X, Webster A, Soria J-C, Wu Y, Nakagawa K, Kim S, Yang J, Ahn M, et al. LBA2_PRGEFITINIB/CHEMOTHERAPY VS CHEMOTHERAPY IN EPIDERMAL GROWTH FACTOR RECEPTOR (EGFR) MUTATION-POSITIVE NON-SMALL-CELL LUNG CANCER (NSCLC) AFTER PROGRESSION ON FIRST-LINE GEFITINIB: THE PHASE III, RANDOMISED IMPRESS STUDY. *Ann Oncol [Internet]* 2014;25. Available from: https://dx.doi.org/10.1093/annonc/mdu438.45

271. Mok TSK, Kim SW, Wu YL, Nakagawa K, Yang JJ, Ahn MJ, Wang J, Yang JCH, Lu Y, Atagi S, Ponce S, Shi X, et al. Gefitinib plus chemotherapy versus chemotherapy in epidermal growth factor receptor mutation-positive non-small-cell lung cancer resistant to first-line gefitinib (IMPRESS): Overall survival and biomarker analyses. *J Clin Oncol* 2017;35:4027–34.

272. Sant M, Francisci S, Minicozzi P, Otter R, Primic-Zakeli M, Gatta G, Rossi S, Baili P, Anderson LA, Holleczek B, Lepage C, Crocetti E, et al. Is Europe doing better in cancer care since the 90s? The latest findings from the EUROCARE-5 study. *Eur J Cancer* 2015;51:S707.

273. Sant M, Minicozzi P, Primic-Žakelj M, Otter R, Francisci S, Gatta G, Berrino F, De Angelis R. Cancer survival in Europe, 1999-2007: Doing better, feeling worse? *Eur J Cancer* 2015;51:2101–3.

274. Atun R, Jaffray D, Barton M, Baumann M, Vikram B, Bray F, Hanna T, Knaul F, Lievens Y, O’Sullivan B, Rodin D, Van Dyke J, et al. Responding to the cancer crisis: Expanding global access to radiotherapy - a Lancet Oncology Commission. *Eur J Cancer* 2015;51:S707–8.

275. Atun R, Jaffray DA, Barton MB, Bray F, Baumann M, Vikram B, Hanna TP, Knaul FM, Lievens Y, Lui TYM, Milosevic M, O’Sullivan B, et al. Expanding global access to radiotherapy. *Lancet Oncol* 2015;16:1153–86.

276. Sharma P, Escudier B, McDermott DF, George S, Hammers HJ, Srinivas S, Tykodi SS, Sosman JA, Procopio G, Plimack ER, Castellano D, Gurney H, et al. CheckMate 025: a randomized, open-label, phase III study of nivolumab (NIVO) versus everolimus (EVE) in advanced renal cell carcinoma (RCC). *Eur J Cancer* 2015;51:S708.

277. Escudier B, Sharma P, McDermott DF, George S, Hammers HJ, Srinivas S, Tykodi SS, Sosman JA, Procopio G, Plimack ER, Castellano D, Gurney H, et al. CheckMate 025 Randomized Phase 3 Study: Outcomes by Key Baseline Factors and Prior Therapy for Nivolumab Versus Everolimus in Advanced Renal Cell Carcinoma [Figure presented]. *Eur Urol* 2017;72:962–71.

278. Choueiri T, Escudier B, Powles T, Mainwaring P, Rini B, Donskov F, Hammers H, Hutson T, Roth B, Peltola K, Lee JL, Heng D, et al. Cabozantinib versus everolimus in patients with advanced renal cell carcinoma: Results of the randomized phase 3 METEOR trial. *Eur J Cancer* 2015;51:S708–9.

279. Choueiri TK, Escudier B, Powles T, Mainwaring PN, Rini BI, Donskov F, Hammers H, Hutson TE, Lee J-L, Peltola K, Roth BJ, Bjarnason GA, et al. Cabozantinib versus Everolimus in Advanced Renal-Cell Carcinoma. *N Engl J Med [Internet]* 2015;373:1814–23. Available from: http://www.nejm.org/doi/10.1056/NEJMoa1510016

280. Vrieling C, Van Werkhoven E, Poortmans P, Struikmans H, Weltens C, Fourquet A, Jager J, Schinagl D, Collette L, Maingon P, Bartelink H. The impact of pathological factors on long-term local control in the EORTC boost no-boost trial. *Eur J Cancer* 2015;51:S709.

281. Vrieling C, Van Werkhoven E, Maingon P, Poortmans P, Weltens C, Fourquet A, Schinagl D, Oei B, Rodenhuis CC, Horiot JC, Struikmans H, Van Limbergen E, et al. Prognostic factors for local control in breast cancer after long-term follow-up in the EORTC boost vs no boost trial: A randomized clinical trial. *JAMA Oncol* 2017;3:42–8.

282. Yao J, Fazio N, Singh S, Buzzoni R, Carnaghi C, Wolin E, Tomasek J, Raderer M, Lahner H, Voi M, Pacaud L, Lincy J, et al. Everolimus in advanced nonfunctional neuroendocrine tumors (NET) of lung or gastrointestinal (GI) origin: Efficacy and safety results from the placebo-controlled, double-blind, multicenter, Phase 3 RADIANT-4 study. *Eur J Cancer* 2015;51:S709–10.

283. Yao JC, Fazio N, Singh S, Buzzoni R, Carnaghi C, Wolin E, Tomasek J, Raderer M, Lahner H, Voi M, Pacaud LB, Rouyrre N, et al. Everolimus for the treatment of advanced, non-functional neuroendocrine tumours of the lung or gastrointestinal tract (RADIANT-4): A randomised, placebo-controlled, phase 3 study. *Lancet* 2016;387:968–77.

284. Strosberg J, Wolin E, Chasen B, Kulke M, Bushnell D, Caplin M, Baum RP, Mittra E, Hobday T, Hendifar A, Oberg K, Sierra ML, et al. 177-Lu-Dotatate significantly improves progression-free survival in patients with midgut neuroendocrine tumours: Results of the phase III NETTER-1 trial. *Eur J Cancer* 2015;51:S710.

285. Strosberg J, El-Haddad G, Wolin E, Hendifar A, Yao J, Chasen B, Mittra E, Kunz PL, Kulke MH, Jacene H, Bushnell D, O’Dorisio TM, et al. Phase 3 Trial of ^177^ Lu-Dotatate for Midgut Neuroendocrine Tumors. *N Engl J Med [Internet]* 2017;376:125–35. Available from: http://www.nejm.org/doi/10.1056/NEJMoa1607427

286. Ophuis CO, Verhoef C, Rutkowski P, Voit C, Cook M, Van Leeuwen P, Testori A, Hoekstra H, Gruenhagen D, Eggermont A, Van Akkooi A. The interval between primary melanoma excision and sentinel node biopsy (SNB) does not affect survival; regardless of SNB status - an EORTC Melanoma Group study. *Eur J Cancer* 2015;51:S711.

287. Oude Ophuis CMC, Verhoef C, Rutkowski P, Powell BWEM, van der Hage JA, van Leeuwen PAM, Voit CA, Testori A, Robert C, Hoekstra HJ, Grünhagen DJ, Eggermont AMM, et al. The interval between primary melanoma excision and sentinel node biopsy is not associated with survival in sentinel node positive patients – An EORTC Melanoma Group study. *Eur J Surg Oncol* 2016;42:1906–13.

288. Stahel RA, Dafni U, Gautschi O, Felip E, Curioni-Fontecedro A, Peters S, Massuti B, Cardenal F, Aix SP, Frueh M, Pless M, Popat S, et al. A phase II trial of erlotinib (E) and bevacizumab (B) in patients with advanced non-small-cell lung cancer (NSCLC) with activating epidermal growth factor receptor (EGFR) mutations with and without T790M mutation. The Spanish Lung Cancer Group (SLCG) and . *Eur J Cancer* 2015;51:S711–2.

289. Rosell R, Dafni U, Felip E, Curioni-Fontecedro A, Gautschi O, Peters S, Massutí B, Palmero R, Aix SP, Carcereny E, Früh M, Pless M, et al. Erlotinib and bevacizumab in patients with advanced non-small-cell lung cancer and activating EGFR mutations (BELIEF): an international, multicentre, single-arm, phase 2 trial. *Lancet Respir Med* 2017;5:435–44.

290. Pietanza MC, Spigel D, Bauer TM, Ready NE, Glisson BS, Morgensztern D, Robert F, Salgia R, Kochendorfer M, Patel M, Strickland DK, Govindan R, et al. Safety, activity, and response durability assessment of single agent rovalpituzumab tesirine, a delta-like protein 3 (DLL3)-targeted antibody drug conjugate (ADC), in small cell lung cancer (SCLC). *Eur J Cancer* 2015;51:S712.

291. Rudin CM, Pietanza MC, Bauer TM, Ready N, Morgensztern D, Glisson BS, Byers LA, Johnson ML, Burris HA, Robert F, Han TH, Bheddah S, et al. Rovalpituzumab tesirine, a DLL3-targeted antibody-drug conjugate, in recurrent small-cell lung cancer: a first-in-human, first-in-class, open-label, phase 1 study. *Lancet Oncol* 2017;18:42–51.

292. Dearnaley D, Syndikus I, Mossop H, Birtle A, Bloomfield D, Cruickshank C, Graham J, Hassan S, Khoo V, Logue J, Mayles H, Money-Kyrle J, et al. 5 year outcomes of a phase III randomised trial of conventional or hypofractionated high dose intensity modulated radiotherapy for prostate cancer (CRUK/06/016): report from the CHHiP Trial Investigators Group. *Eur J Cancer* 2015;51:S712.

293. Dearnaley D, Syndikus I, Mossop H, Khoo V, Birtle A, Bloomfield D, Graham J, Kirkbride P, Logue J, Malik Z, Money-Kyrle J, O’Sullivan JM, et al. Conventional versus hypofractionated high-dose intensity-modulated radiotherapy for prostate cancer: 5-year outcomes of the randomised, non-inferiority, phase 3 CHHiP trial. *Lancet Oncol* 2016;17:1047–60.

294. Sullivan R, Olusegun IA, Anderson BO, Audisio R, Autier P, Aggarwal A, Balch C, Brennan M, Dare A, D’Cruz A, Eggermont A, Fleming K, et al. Delivering safe and affordable cancer surgery to all - a Lancet Oncology Commission. *Eur J Cancer* 2015;51:S713.

295. Sullivan R, Alatise OI, Anderson BO, Audisio R, Autier P, Aggarwal A, Balch C, Brennan MF, Dare A, D’Cruz A, Eggermont AMM, Fleming K, et al. Global cancer surgery: Delivering safe, affordable, and timely cancer surgery. *Lancet Oncol [Internet]* 2015;16:1193–224. Available from: https://doi.org/10.1016/S1470-2045(15)00223-5

296. Carducci M, Armstrong A, Pili R, Ng S, Huddart R, Agarwal N, Khvorostenko D, Lyulko O, Brize A, Vogelzang NJ, Delva R, Harza M, et al. A phase 3, randomized, double-blind, placebo-controlled study of tasquinimod (TASQ) in men with metastatic castrate resistant prostate cancer (mCRPC). *Eur J Cancer* 2015;51:S713–4.

297. Sternberg C, Armstrong A, Pili R, Ng S, Huddart R, Agarwal N, Khvorostenko D, Lyulko O, Brize A, Vogelzang N, Delva R, Harza M, et al. Randomized, double-blind, placebo-controlled phase III study of tasquinimod in men with metastatic castration-resistant prostate cancer. *J Clin Oncol* 2016;34:2636–43.

298. Sparano J, Gray R, Zujewski JA, Makower D, Pritchard K, Albain K, Hayes D, Geyer C, Dees C, Perez E, Keane M, Vallejos C, et al. Prospective trial of endocrine therapy alone in patients with estrogen-receptor positive, HER2-negative, node-negative breast cancer: Results of the TAILORx low risk registry. *Eur J Cancer* 2015;51:S714.

299. Hortobagyi GN, Stemmer SM, Burris HA, Yap YS, Sonke GS, Paluch-Shimon S, Campone M, Blackwell K, Andre F, Winer EP, Janni W, Verma S, et al. First-line ribociclib plus letrozole for postmenopausal women with hormone receptor-positive (HR+), HER2-negative (HER2-), advanced breast cancer (ABC). *Ann Oncol* 2016;27.

300. Hortobagyi GN, Stemmer SM, Burris HA, Yap Y-S, Sonke GS, Paluch-Shimon S, Campone M, Blackwell KL, André F, Winer EP, Janni W, Verma S, et al. Ribociclib as First-Line Therapy for HR-Positive, Advanced Breast Cancer. *N Engl J Med [Internet]* 2016;375:1738–48. Available from: http://www.nejm.org/doi/10.1056/NEJMoa1609709

301. Eggermont AMM, Chiarion-Sileni V, Grob J-J, Dummer R, Wolchok JD, Schmidt H, Hamid O, Robert C, Ascierto PA, Richards JM, Lebbe C, Ferraresi V, et al. Ipilimumab (IPI) vs placebo (PBO) after complete resection of stage III melanoma: final overall survival results from the EORTC 18071 randomized, double-blind, phase 3 trial. *Ann Oncol* 2016;27.

302. Eggermont AMM, Chiarion-Sileni V, Grob J-J, Dummer R, Wolchok JD, Schmidt H, Hamid O, Robert C, Ascierto PA, Richards JM, Lebbé C, Ferraresi V, et al. Prolonged Survival in Stage III Melanoma with Ipilimumab Adjuvant Therapy. *N Engl J Med [Internet]* 2016;375:1845–55. Available from: http://www.nejm.org/doi/10.1056/NEJMoa1611299

303. Mirza MR, Monk BJ, Oza A, Mahner S, Redondo A, Fabbro M, Ledermann J, Lorusso D, Vergote IB, Rosengarten O, Berek J, Herrstedt J, et al. A randomized, double-blind phase 3 trial of maintenance therapy with niraparib vs placebo in patients with platinum-sensitive recurrent ovarian cancer (ENGOT-OV16/NOVA trial). *Ann Oncol* 2016;27.

304. Mirza MR, Monk BJ, Herrstedt J, Oza AM, Mahner S, Redondo A, Fabbro M, Ledermann JA, Lorusso D, Vergote I, Ben-Baruch NE, Marth C, et al. Niraparib Maintenance Therapy in Platinum-Sensitive, Recurrent Ovarian Cancer. *N Engl J Med [Internet]* 2016;375:2154–64. Available from: http://www.nejm.org/doi/10.1056/NEJMoa1611310

305. Harrington K, Ferris RL, Shaw J, Taylor F, Derosa M, Turner-Bowker D, Morrissey L, Cocks K, Kiyota N, Gillison M, Guigay J. Patient-reported outcomes (PROs) in recurrent or metastatic (R/M) squamous cell carcinoma of the head and neck (SCCHN) treated with nivolumab (nivo) or investigator’s choice (IC): CheckMate 141. *Ann Oncol* 2016;27.

306. Harrington KJ, Ferris RL, Blumenschein Jr. G, Colevas AD, Fayette J, Licitra L, Kasper S, Even C, Vokes EE, Worden F, Saba NF, Kiyota N, et al. Nivolumab versus standard, single-agent therapy of investigator’s choice in recurrent or metastatic squamous cell carcinoma of the head and neck (CheckMate 141): health-related quality-of-life results from a randomised, phase 3 trial. *Lancet Oncol* 2017;18:1104–15.

307. Langer C, Gaddgeel SM, Borghaei H, Papadimitrakopoulou VA, Patnaik A, Powell S, Gentzler RD, Martins RG, Stevenson JP, Jalal SI, Panwalkar A, Yang JC-H, et al. Randomized, phase 2 study of carboplatin and pemetrexed with or without pembrolizumab as first-line therapy for advanced NSCLC: KEYNOTE-021 cohort G. *Ann Oncol* 2016;27.

308. Langer CJ, Gadgeel SM, Borghaei H, Papadimitrakopoulou VA, Patnaik A, Powell SF, Gentzler RD, Martins RG, Stevenson JP, Jalal SI, Panwalkar A, Yang JCH, et al. Carboplatin and pemetrexed with or without pembrolizumab for advanced, non-squamous non-small-cell lung cancer: a randomised, phase 2 cohort of the open-label KEYNOTE-021 study. *Lancet Oncol* 2016;17:1497–508.

309. Reck M, Horn L, Novello S, Barlesi F, Albert I, Juhasz E, Chung J, Fritsch A, Drews U, Rutstein M, Wagner A, Govindan R. Phase II study of roniciclib in combination with cisplatin/etoposide or carboplatin/etoposide as first-line therapy in subjects with extensive-disease small cell lung cancer (ED-SCLC). *Ann Oncol* 2016;27.

310. Reck M, Rodriguez-Abreu D, Robinson AG, Hui R, Csoszi T, Fulop A, Gottfried M, Peled N, Tafreshi A, Cuffe S, O’Brien M, Rao S, et al. Pembrolizumab versus Chemotherapy for PD-L1-Positive Non-Small-Cell Lung Cancer. *N Engl J Med* 2016;375:1823–33.

311. Socinski M, Creelan B, Horn L, Reck M, Paz-Ares L, Steins M, Felip E, van den Heuvel M, Ciuleanu TE, Badin F, Ready N, Hiltermann TJN, et al. CheckMate 026: A phase 3 trial of nivolumab vs investigator’s choice (IC) of platinum-based doublet chemotherapy (PT-DC) as first-line therapy for stage iv/recurrent programmed death ligand 1 (PD-L1)-positive NSCLC. *Ann Oncol* 2016;27.

312. Carbone DP, Reck M, Paz-Ares L, Creelan B, Horn L, Steins M, Felip E, van den Heuvel MM, Ciuleanu T-E, Badin F, Ready N, Hiltermann TJN, et al. First-Line Nivolumab in Stage IV or Recurrent Non–Small-Cell Lung Cancer. *N Engl J Med [Internet]* 2017;376:2415–26. Available from: http://www.nejm.org/doi/10.1056/NEJMoa1613493

313. Barlesi F, Park K, Ciardiello F, von Pawel J, Gadgeel S, Hida T, Kowalski D, Dols MC, Cortinovis D, Leach J, Polikoff J, Gandara D, et al. Primary analysis from OAK, a randomized phase III study comparing atezolizumab with docetaxel in 2L/3L NSCLC. *Ann Oncol* 2016;27.

314. Rittmeyer A, Barlesi F, Waterkamp D, Park K, Ciardiello F, von Pawel J, Gadgeel SM, Hida T, Kowalski DM, Dols MC, Cortinovis DL, Leach J, et al. Atezolizumab versus docetaxel in patients with previously treated non-small-cell lung cancer (OAK): a phase 3, open-label, multicentre randomised controlled trial. *Lancet* 2017;389:255–65.

315. Gronchi A, Ferrari S, Quagliuolo V, Broto JM, Lopez-Pousa A, Grignani G, Ferraresi V, Blay J-Y, Rutkowski P, Merlo FD, Marchesi E, Ledesma P, et al. sarcomaFull-dose neoadjuvant anthracycline + ifosfamide chemotherapy is associated with a relapse free survival (RFS) and overall survival (OS) benefit in localized high-risk adult soft tissue sarcomas (STS) of the extremities and trunk wall: Interim anal. *Ann Oncol [Internet]* 2016;27. Available from: http://academic.oup.com/annonc/article/doi/10.1093/annonc/mdw435.52/2800561/sarcomaFulldose-neoadjuvant-anthracycline

316. Gronchi A, Ferrari S, Quagliuolo V, Broto JM, Pousa AL, Grignani G, Basso U, Blay JY, Tendero O, Beveridge RD, Ferraresi V, Lugowska I, et al. Histotype-tailored neoadjuvant chemotherapy versus standard chemotherapy in patients with high-risk soft-tissue sarcomas (ISG-STS 1001): an international, open-label, randomised, controlled, phase 3, multicentre trial. *Lancet Oncol* 2017;18:812–22.

317. Beer TM, Hotte SJ, Saad F, Alekseev B, Matveev V, Fléchon A, Gravis G, Joly F, Chi KN, Malik Z, Blumenstein B, Stewart PS, et al. Custirsen (OGX-011) combined with cabazitaxel and prednisone versus cabazitaxel and prednisone alone in patients with metastatic castration-resistant prostate cancer previously treated with docetaxel (AFFINITY): a randomised, open-label, international, ph. *Lancet Oncol* 2017;18:1532–42.

318. Choueiri TK, Halabi S, Sanford B, Hahn O, Michaelson MD, Walsh M, Olencki T, Picus J, Small EJ, Dakhil S, George D, Morris MJ. CABOzantinib versus SUNitinib (CABOSUN) as initial targeted therapy for patients with metastatic renal cell carcinoma (mRCC) of poor and intermediate risk groups: Results from ALLIANCE A031203 trial. *Ann Oncol* 2016;27.

319. Choueiri TK, Halabi S, Sanford BL, Hahn O, Michaelson MD, Walsh MK, Feldman DR, Olencki T, Picus J, Small EJ, Dakhil S, George DJ, et al. Cabozantinib versus sunitinib as initial targeted therapy for patients with metastatic renal cell carcinoma of poor or intermediate risk: The alliance A031203 CABOSUN trial. *J Clin Oncol* 2017;35:591–7.

320. Ravaud A, Motzer RJ, Pandha HS, Staehler M, George D, Pantuck AJ, Patel A, Chang Y-H, Escudier B, Donskov F, Magheli A, Carteni G, et al. Phase III trial of sunitinib (SU) vs placebo (PBO) as adjuvant treatment for high-risk renal cell carcinoma (RCC) after nephrectomy (S-TRAC). *Ann Oncol* 2016;27.

321. Ravaud A, Motzer RJ, Pandha HS, George DJ, Pantuck AJ, Patel A, Chang Y-H, Escudier B, Donskov F, Magheli A, Carteni G, Laguerre B, et al. Adjuvant Sunitinib in High-Risk Renal-Cell Carcinoma after Nephrectomy. *N Engl J Med* 2016;375:2246–54.

322. Paz-Ares L, Villegas A, Daniel D, Baz D V, Murakami S, Hui R, Yokoi T, Chiappori A, Lee KH, de Wit M, Cho BC, Bourhaba M, et al. PACIFIC: A double-blind, placebo-controlled phase III study of durvalumab after chemoradiation therapy (CRT) in patients with stage III, locally advanced, unresectable NSCLC. *Ann Oncol* 2017;28.

323. Antonia SJ, Villegas A, Daniel D, Vicente D, Murakami S, Hui R, Yokoi T, Chiappori A, Lee KH, de Wit M, Cho BC, Bourhaba M, et al. Durvalumab after Chemoradiotherapy in Stage III Non-Small-Cell Lung Cancer. *N Engl J Med* 2017;377:1919–29.

324. Westeel V, Barlesi F, Foucher P, Lafitte J-J, Domas J, Girard P, Tredaniel J, Wislez M, Dumont P, Quoix E, Raffy O, Braun D, et al. Results of the phase III IFCT-0302 trial assessing minimal versus CT-scan-based follow-up for completely resected non-small cell lung cancer (NSCLC). *Ann Oncol* 2017;28.

325. Ramalingam S, Reungwetwattana T, Chewaskulyong B, Dechaphunkul A, Lee KH, Imamura F, Nogami N, Ohe Y, Cheng Y, Cho BC, Cho EK, Vansteenkiste JF, et al. Osimertinib vs standard of care (SoC) EGFR-TKI as first-line therapy in patients (pts) with EGFRm advanced NSCLC: FLAURA. *Ann Oncol* 2017;28.

326. Soria J-C, Ohe Y, Vansteenkiste J, Reungwetwattana T, Chewaskulyong B, Lee KH, Dechaphunkul A, Imamura F, Nogami N, Kurata T, Okamoto I, Zhou C, et al. Osimertinib in Untreated EGFR-Mutated Advanced Non–Small-Cell Lung Cancer. *N Engl J Med [Internet]* 2018;378:113–25. Available from: https://doi.org/10.1056/NEJMoa1713137

327. Di Leo A, Toi M, Campone M, Sohn J, Paluch-Shimon S, Huober J, Park IH, Tredan O, Chen S-C, Manso L, Freedman O, Jaliffe GG, et al. MONARCH 3: Abemaciclib as initial therapy for patients with HR+/HER2-advanced breast cancer. *Ann Oncol* 2017;28.

328. Goetz MP, Toi M, Campone M, Trédan O, Bourayou N, Sohn J, Park IH, Paluch-Shimon S, Huober J, Chen SC, Manso L, Barriga S, et al. MONARCH 3: Abemaciclib as initial therapy for advanced breast cancer. *J Clin Oncol* 2017;35:3638–46.

329. Gupta S, Parab P, Kerkar R, Mahantshetty U, Maheshwari A, Sastri S, Engineer R, Hawaldar R, Ghosh J, Gulia S, Godbole S, Kumar N, et al. Neoadjuvant chemotherapy followed by surgery (NACT-surgery) versus concurrent cisplatin and radiation therapy (CTRT) in patients with stage IB2 to IIB squamous carcinoma of cervix: A randomized controlled trial (RCT). *Ann Oncol* 2017;28.

330. Gupta S, Maheshwari A, Parab P, Mahantshetty U, Hawaldar R, Sastri S, Kerkar R, Engineer R, Tongaonkar H, Ghosh J, Gulia S, Kumar N, et al. Neoadjuvant Chemotherapy Followed by Radical Surgery Versus Concomitant Chemotherapy and Radiotherapy in Patients With Stage IB2, IIA, or IIB Squamous Cervical Cancer: A Randomized Controlled Trial. *J Clin Oncol* 2018;36:1548–55.

331. Petrylak DP, Chi KN, Drakaki A, Sternberg CN, de Wit R, Nishiyama H, Yu EY, Castellano D, Hussain S, Percent IJ, Flechon A, Bamias A, et al. RANGE: A randomized, double-blind, placebo-controlled phase 3 study of docetaxel (DOC) with or without ramucirumab (RAM) in platinum-refractory advanced or metastatic urothelial carcinoma. *Ann Oncol* 2017;28.

332. Petrylak D, de Wit R, Chi KN, Drakaki A, Sternberg CN, Nishiyama H, Castellano D, Hussain S, Fléchon A, Bamias A, Yu EY, van der Heijden MS, et al. Ramucirumab plus docetaxel versus placebo plus docetaxel in patients with locally advanced or metastatic urothelial carcinoma after platinum-based therapy (RANGE): a randomised, double-blind, phase 3 trial. *Lancet* 2017;390:2266–77.

333. Escudier B, Tannir NM, McDermott DF, Frontera OA, Melichar B, Plimack ER, Barthelemy P, George S, Neiman V, Porta C, Choueiri TK, Powles T, et al. CheckMate 214: Efficacy and safety of nivolumab plus ipilimumab (N plus I) v sunitinib (S) for treatment-naive advanced or metastatic renal cell carcinoma (mRCC), including IMDC risk and PD-L1 expression subgroups. *Ann Oncol* 2017;28.

334. Weber J, Mandala M, Del Vecchio M, Gogas HJ, Arance AM, Cowey CL, Dalle S, Schenker M, Chiarion-Sileni V, Marquez-Rodas I, Grob J-J, Butler MO, et al. Adjuvant Nivolumab versus Ipilimumab in Resected Stage III or IV Melanoma. *N Engl J Med [Internet]* 2017;377:NEJMoa1709030. Available from: http://www.nejm.org/doi/10.1056/NEJMoa1709030

335. Lewis K, Maio M, Demidov L, Mandala M, Ascierto PA, Herbert C, Mackiewicz A, Rutkowski P, Guminski A, Goodman G, Simmons B, Ye C, et al. BRIM8: a randomized, double-blind, placebo-controlled study of adjuvant vemurafenib in patients (pts) with completely resected, BRAFV600+melanoma at high risk for recurrence. *Ann Oncol* 2017;28.

336. Maio M, Lewis K, Demidov L, Mandalà M, Bondarenko I, Ascierto PA, Herbert C, Mackiewicz A, Rutkowski P, Guminski A, Goodman GR, Simmons B, et al. Adjuvant vemurafenib in resected, BRAFV600 mutation-positive melanoma (BRIM8): a randomised, double-blind, placebo-controlled, multicentre, phase 3 trial. *Lancet Oncol* 2018;19:510–20.

337. Hauschild A, Santinami M, Long G V, Atkinson V, Mandala M, Sileni VC, Nyakas MS, Dutriaux C, Haydon A, Robert C, Mortier L, Schachter J, et al. COMBI-AD: Adjuvant dabrafenib (D) plus trametinib (T) for resected stage III BRAF V600E/K-mutant melanoma. *Ann Oncol* 2017;28.

338. Long G V, Hauschild A, Santinami M, Atkinson V, Mandala M, Chiarion-Sileni V, Larkin J, Nyakas M, Dutriaux C, Haydon A, Robert C, Mortier L, et al. Adjuvant Dabrafenib plus Trametinib in Stage III BRAF-Mutated Melanoma. *N Engl J Med* 2017;377:1813–23.

339. Weber J, Mandala M, Del Vecchio M, Gogas H, Arance AM, Cowey LC, Dalle S, Schenker M, Chiarion-Sileni V, Marquez-Rodas I, Grob J-J, Butler M, et al. Adjuvant therapy with nivolumab (NIVO) versus ipilimumab (IPI) after complete resection of stage III/IV melanoma: A randomized, double-blind, phase 3 trial (CheckMate 238). *Ann Oncol* 2017;28.

340. Schmid P, Wright GS, Husain A, Henschel V, Molinero L, Funke R, Chui SY, Winer EP, Loi S, Emens LA, Adams S, Rugo HS, et al. LBA1_PRIMpassion130: Results from a global, randomised, double-blind, phase III study of atezolizumab (atezo) + nab-paclitaxel (nab-P) vs placebo + nab-P in treatment-naive, locally advanced or metastatic triple-negative breast cancer (mTNBC). *Ann Oncol [Internet]* 2018;29. Available from: https://dx.doi.org/10.1093/annonc/mdy424.008

341. Schmid P, Adams S, Rugo HS, Schneeweiss A, Barrios CH, Iwata H, Diéras V, Hegg R, Im S-A, Shaw Wright G, Henschel V, Molinero L, et al. Atezolizumab and Nab-Paclitaxel in Advanced Triple-Negative Breast Cancer. *N Engl J Med [Internet]* 2018;379:NEJMoa1809615. Available from: http://www.nejm.org/doi/10.1056/NEJMoa1809615

342. Cristofanilli M, Verma S, Iwata H, Harbeck N, Loibl S, André F, Puyana Theall K, Huang X, Giorgetti C, Huang Bartlett C, Turner NC, Slamon DJ, et al. LBA2_PROverall survival (OS) with palbociclib plus fulvestrant in women with hormone receptor-positive (HR+), human epidermal growth factor receptor 2-negative (HER2−) advanced breast cancer (ABC): Analyses from PALOMA-3. *Ann Oncol [Internet]* 2018;29. Available from: https://dx.doi.org/10.1093/annonc/mdy424.009

343. Turner NC, Slamon DJ, Ro J, Bondarenko I, Im S-A, Masuda N, Colleoni M, DeMichele A, Loi S, Verma S, Iwata H, Harbeck N, et al. Overall Survival with Palbociclib and Fulvestrant in Advanced Breast Cancer. *N Engl J Med [Internet]* 2018;379:NEJMoa1810527. Available from: http://www.nejm.org/doi/10.1056/NEJMoa1810527

344. André F, Ciruelos EM, Rubovszky G, Campone M, Loibl S, Rugo HS, Iwata H, Conte P, Mayer IA, Kaufman B, Yamashita T, Lu Y-S, et al. LBA3_PRAlpelisib (ALP) + fulvestrant (FUL) for advanced breast cancer (ABC): Results of the phase III SOLAR-1 trial. *Ann Oncol [Internet]* 2018;29:mdy424.010. Available from: http://dx.doi.org/10.1093/annonc/mdy424.010

345. André F, Ciruelos E, Rubovszky G, Campone M, Loibl S, Rugo HS, Iwata H, Conte P, Mayer IA, Kaufman B, Yamashita T, Lu Y-S, et al. Alpelisib for PIK3CA -Mutated, Hormone Receptor–Positive Advanced Breast Cancer . *N Engl J Med* 2019;380:1929–40.

346. Jiang Z, Li W, Hu X, Zhang Q, Sun T, Cui S, Wang S, Ouyang Q, Yin Y, Geng C, Tong Z, Cheng Y, et al. 283O_PRPhase III trial of chidamide, a subtype-selective histone deacetylase (HDAC) inhibitor, in combination with exemestane in patients with hormone receptor-positive advanced breast cancer. *Ann Oncol [Internet]* 2018;29:mdy424.011. Available from: https://academic.oup.com/annonc/article/doi/10.1093/annonc/mdy424.011/5141531

347. Jiang Z, Li W, Hu X, Zhang Q, Sun T, Cui S, Wang S, Ouyang Q, Yin Y, Geng C, Tong Z, Cheng Y, et al. Tucidinostat plus exemestane for postmenopausal patients with advanced, hormone receptor-positive breast cancer (ACE): a randomised, double-blind, placebo-controlled, phase 3 trial. *Lancet Oncol* 2019;20:806–15.

348. Hoyle AP, Ali SA, Jones R, Matheson D, Mason MD, Russell M, Clarke NW, James ND, Parker CC, Ritchie A, Cook AD, Gilson CE, et al. LBA4Effects of abiraterone acetate plus prednisone/prednisolone in high and low risk metastatic hormone sensitive prostate cancer. *Ann Oncol [Internet]* 2018;29. Available from: https://dx.doi.org/10.1093/annonc/mdy424.033

349. Parker CC, Lester J, Mason MD, Millman R, Gillessen S, Eswar C, Gale J, Sheehan D, Tran A, James ND, Brawley C, Gilson CE, et al. LBA5_PRRadiotherapy (RT) to the primary tumour for men with newly-diagnosed metastatic prostate cancer (PCa): Survival results from STAMPEDE. *Ann Oncol [Internet]* 2018;29. Available from: https://dx.doi.org/10.1093/annonc/mdy424.034

350. Parker CC, James ND, Brawley CD, Clarke NW, Hoyle AP, Ali A, Ritchie AWS, Attard G, Chowdhury S, Cross W, Dearnaley DP, Gillessen S, et al. Radiotherapy to the primary tumour for newly diagnosed, metastatic prostate cancer (STAMPEDE): a randomised controlled phase 3 trial. *Lancet* 2018;392:2353–66.

351. Motzer RJ, Penkov K, Haanen JBAG, Rini BI, Albiges L, Campbell MT, Kollmannsberger CK, Negrier S, Uemura M, Lee JL, Gurney H, Berger R, et al. LBA6_PRJAVELIN renal 101: A randomized, phase III study of avelumab + axitinib vs sunitinib as first-line treatment of advanced renal cell carcinoma (aRCC). *Ann Oncol [Internet]* 2018;29:mdy424.036. Available from: https://academic.oup.com/annonc/article/doi/10.1093/annonc/mdy424.036/5141753

352. Motzer RJ, Penkov K, Haanen J, Rini B, Albiges L, Campbell MT, Venugopal B, Kollmannsberger C, Negrier S, Uemura M, Lee JL, Vasiliev A, et al. Avelumab plus Axitinib versus Sunitinib for Advanced Renal-Cell Carcinoma. *N Engl J Med* 2019;380:1103–15.

353. Moore KN, Sonke GS, Gourley C, Banerjee S, Oza AM, González-Martín A, Aghajanian C, Bradley W, Lowe ES, Bloomfield R, DiSilvestro P, Colombo N, et al. LBA7_PRMaintenance olaparib following platinum-based chemotherapy in newly diagnosed patients (pts) with advanced ovarian cancer (OC) and a BRCA1/2 mutation (BRCAm): Phase III SOLO1 trial. *Ann Oncol [Internet]* 2018;29. Available from: https://dx.doi.org/10.1093/annonc/mdy424.041

354. Moore K, Colombo N, Scambia G, Kim B-G, Oaknin A, Friedlander M, Lisyanskaya A, Floquet A, Leary A, Sonke GS, Gourley C, Banerjee S, et al. Maintenance Olaparib in Patients with Newly Diagnosed Advanced Ovarian Cancer. *N Engl J Med [Internet]* 2018;379:NEJMoa1810858. Available from: http://www.nejm.org/doi/10.1056/NEJMoa1810858

355. Burtness B, Bratland Å, Fuereder T, Hughes BGM, Mesia R, Ngamphaiboon N, Rordorf T, Wan Ishak WZ, Roy A, Jin F, Cheng J, Rischin D, et al. LBA8_PRKEYNOTE-048: Phase III study of first-line pembrolizumab (P) for recurrent/metastatic head and neck squamous cell carcinoma (R/M HNSCC). *Ann Oncol [Internet]* 2018;29. Available from: https://dx.doi.org/10.1093/annonc/mdy424.045

356. Kong A, Mehanna H, Fulton-Lieuw T, Al Booz H, Moleron R, Brennan S, Aynsley E, Chan A, Srinivasan D, Buter J, Hartley A, Dunn J, et al. LBA9_PRCetuximab versus cisplatin in patients with HPV-positive, low risk oropharyngeal cancer, receiving radical radiotherapy. *Ann Oncol [Internet]* 2018;29. Available from: https://dx.doi.org/10.1093/annonc/mdy424.046

357. Mehanna H, Robinson M, Hartley A, Kong A, Foran B, Fulton-Lieuw T, Dalby M, Mistry P, Sen M, O’Toole L, Al Booz H, Dyker K, et al. Radiotherapy plus cisplatin or cetuximab in low-risk human papillomavirus-positive oropharyngeal cancer (De-ESCALaTE HPV): an open-label randomised controlled phase 3 trial. *Lancet (London, England)* 2019;393:51–60.

358. Zhou C, Lu Y, Kim S-W, Reungwetwattana T, Zhou J, Zhang Y, He J, Yang J-J, Cheng Y, Lee SH, Bu L, Xu T, et al. LBA10Primary results of ALESIA: A randomised, phase III, open-label study of alectinib vs crizotinib in Asian patients with treatment-naïve ALK+ advanced NSCLC. *Ann Oncol [Internet]* 2018;29:mdy424.062. Available from: http://dx.doi.org/10.1093/annonc/mdy424.062

359. Zhou C, Kim SW, Reungwetwattana T, Zhou J, Zhang Y, He J, Yang JJ, Cheng Y, Lee SH, Bu L, Xu T, Yang L, et al. Alectinib versus crizotinib in untreated Asian patients with anaplastic lymphoma kinase-positive non-small-cell lung cancer (ALESIA): a randomised phase 3 study. *Lancet Respir Med* 2019;7:437–46.
